# Effects of water quality, sanitation, handwashing, and nutritional interventions on diarrhoea and child growth in rural Bangladesh: a cluster randomised controlled trial

**DOI:** 10.1016/S2214-109X(17)30490-4

**Published:** 2018-01-29

**Authors:** Stephen P Luby, Mahbubur Rahman, Benjamin F Arnold, Leanne Unicomb, Sania Ashraf, Peter J Winch, Christine P Stewart, Farzana Begum, Faruqe Hussain, Jade Benjamin-Chung, Elli Leontsini, Abu M Naser, Sarker M Parvez, Alan E Hubbard, Audrie Lin, Fosiul A Nizame, Kaniz Jannat, Ayse Ercumen, Pavani K Ram, Kishor K Das, Jaynal Abedin, Thomas F Clasen, Kathryn G Dewey, Lia C Fernald, Clair Null, Tahmeed Ahmed, John M Colford

**Affiliations:** a Division of Infectious Diseases and Geographic Medicine, Stanford University, Stanford, CA, USA; b International Centre for Diarrhoeal Disease Research, Dhaka, Bangladesh; c School of Public Health University of California Berkeley, Berkeley, CA, USA; d Department of International Health, Johns Hopkins Bloomberg School of Public Health, Baltimore, MD, USA; e Department of Nutrition, University of California Davis, Davis, CA, USA; f School of Public Health and Health Professions, University of Buffalo, Buffalo, NY, USA; g Rollins School of Public Health, Emory University, Atlanta, GA, USA

## Abstract

**Background:**

Diarrhoea and growth faltering in early childhood are associated with subsequent adverse outcomes. We aimed to assess whether water quality, sanitation, and handwashing interventions alone or combined with nutrition interventions reduced diarrhoea or growth faltering.

**Methods:**

The WASH Benefits Bangladesh cluster-randomised trial enrolled pregnant women from villages in rural Bangladesh and evaluated outcomes at 1-year and 2-years' follow-up. Pregnant women in geographically adjacent clusters were block-randomised to one of seven clusters: chlorinated drinking water (water); upgraded sanitation (sanitation); promotion of handwashing with soap (handwashing); combined water, sanitation, and handwashing; counselling on appropriate child nutrition plus lipid-based nutrient supplements (nutrition); combined water, sanitation, handwashing, and nutrition; and control (data collection only). Primary outcomes were caregiver-reported diarrhoea in the past 7 days among children who were in utero or younger than 3 years at enrolment and length-for-age *Z* score among children born to enrolled pregnant women. Masking was not possible for data collection, but analyses were masked. Analysis was by intention to treat. This trial is registered at ClinicalTrials.gov, number NCC01590095.

**Findings:**

Between May 31, 2012, and July 7, 2013, 5551 pregnant women in 720 clusters were randomly allocated to one of seven groups. 1382 women were assigned to the control group; 698 to water; 696 to sanitation; 688 to handwashing; 702 to water, sanitation, and handwashing; 699 to nutrition; and 686 to water, sanitation, handwashing, and nutrition. 331 (6%) women were lost to follow-up. Data on diarrhoea at year 1 or year 2 (combined) were available for 14 425 children (7331 in year 1, 7094 in year 2) and data on length-for-age *Z* score in year 2 were available for 4584 children (92% of living children were measured at year 2). All interventions had high adherence. Compared with a prevalence of 5·7% (200 of 3517 child weeks) in the control group, 7-day diarrhoea prevalence was lower among index children and children under 3 years at enrolment who received sanitation (61 [3·5%] of 1760; prevalence ratio 0·61, 95% CI 0·46–0·81), handwashing (62 [3·5%] of 1795; 0·60, 0·45–0·80), combined water, sanitation, and handwashing (74 [3·9%] of 1902; 0·69, 0·53–0·90), nutrition (62 [3·5%] of 1766; 0·64, 0·49–0·85), and combined water, sanitation, handwashing, and nutrition (66 [3·5%] of 1861; 0·62, 0·47–0·81); diarrhoea prevalence was not significantly lower in children receiving water treatment (90 [4·9%] of 1824; 0·89, 0·70–1·13). Compared with control (mean length-for-age *Z* score −1·79), children were taller by year 2 in the nutrition group (mean difference 0·25 [95% CI 0·15–0·36]) and in the combined water, sanitation, handwashing, and nutrition group (0·13 [0·02–0·24]). The individual water, sanitation, and handwashing groups, and combined water, sanitation, and handwashing group had no effect on linear growth.

**Interpretation:**

Nutrient supplementation and counselling modestly improved linear growth, but there was no benefit to the integration of water, sanitation, and handwashing with nutrition. Adherence was high in all groups and diarrhoea prevalence was reduced in all intervention groups except water treatment. Combined water, sanitation, and handwashing interventions provided no additive benefit over single interventions.

**Funding:**

Bill & Melinda Gates Foundation.

## Introduction

Over 200 million children born in low-income countries are at risk of not reaching their development potential.^1^ Poor linear growth in early childhood is a marker for chronic deprivation that is associated with increased mortality, impaired cognitive development, and reduced adult income.^2^ Nutrition-specific interventions have been shown to improve child growth but they have only corrected a small part of the total growth deficit.^3^

**Research in context**

**Evidence before this study**

Although malnutrition and diarrhoeal disease in children have been known for decades to impair child health and growth, there is little evidence on interventions that are successful at improving growth and reducing diarrhoea. Several observational analyses noted positive associations between improvements in water, sanitation, and handwashing conditions and child growth, but at the time this study was conceived there were no published randomised controlled trials specifically powered to evaluate the effect of such interventions on child growth as a primary outcome. Subsequent published trials of sanitation interventions have reported mixed results. Systematic reviews of complementary feeding interventions have reported small but significant improvements in child growth. More recent evidence from lipid-based nutrient supplementation trials has been mostly consistent with these earlier systematic reviews. Chronic enteric infection might affect children's capacity to respond to nutrients; however, we found no published studies comparing the effect on child growth of nutritional interventions alone versus nutritional interventions plus water, sanitation, and handwashing interventions. Although many programmatic interventions target multiple pathways of enteric pathogen transmission, systematic reviews have found no greater reduction in diarrhoea with combined versus single water, sanitation, and handwashing interventions. There is little direct evidence comparing interventions that target a single versus multiple pathways. Only three randomised controlled trials compared single versus combined interventions in comparable populations at the same time. None of these trials found a significant reduction in diarrhoea among children younger than 5 years who received combined versus the most effective single intervention.

**Added value of this study**

This trial was designed to compare the effects of individual and combined water quality, sanitation, hygiene, and nutrient supplementation plus infant and young child feeding counselling interventions on diarrhoea and growth when given to infants and young children in a setting where child growth faltering was common. The trial had high intervention adherence, low attrition, and ample statistical power to detect small effects. Children receiving interventions with nutritional components had small growth benefits compared with those in the control cluster. Water quality, sanitation, and handwashing interventions did not improve child growth, neither when delivered alone nor when combined with the nutritional interventions. Children receiving sanitation, handwashing, nutrition, and combined interventions had less reported diarrhoea. Combined interventions showed no additional reduction in diarrhoea beyond single interventions.

**Implications of all the available evidence**

The modest improvements observed in growth faltering with nutritional supplementation and counselling are consistent with other trials that report similar levels of efficacy in some contexts. By contrast to observational studies that report an association between growth faltering and water, sanitation, and hygiene assessments, this intervention trial provides no evidence that household drinking water quality, sanitation, or handwashing interventions consistently improve growth. This trial further supports findings from smaller trials that combined individual water, sanitation, and handwashing interventions are not consistently more effective in the prevention of diarrhoea than are single interventions.

Environmental enteric dysfunction is an abnormality of gut function that might explain why most nutrition interventions fail to normalise early childhood growth.^4^ Environmental contaminants are thought to induce the chronic intestinal inflammation, loss of villous surface area, and impaired barrier function that combine to impair food and nutrient uptake. Several observational studies find that children living in communities where most people have access to a toilet are less likely to be stunted than are children who live in communities where open defecation is more common.^5^ Intervention trials to reduce exposure to human faeces can resolve questions of confounding in the relationship between toilet access and child growth and evaluate potential interventions. Improvements to drinking water quality, sanitation, and handwashing might improve the effectiveness of nutrition interventions and thereby help to tackle a larger portion of the observed growth deficit.

In addition to asymptomatic infections and subclinical changes to the gut, episodes of symptomatic diarrhoea accounted for about 500 000 deaths of children younger than 5 years in 2015.^6^ Approaches to reduce diarrhoea include treated drinking water, improved sanitation, and increased handwashing with soap. Although funding a single intervention for a larger population might improve health more than multiple interventions that target a smaller population, data to inform such decisions are scarce.

Interventions that combine nutrition and water, sanitation, and handwashing might provide multiple benefits to children, but there is little evidence that directly compares the effects of individual and combined interventions on diarrhoea and growth of young children.^7^, ^8^

We aimed to investigate whether individual water, sanitation, handwashing, or nutrition interventions can reduce linear growth faltering; to assess whether combined water, sanitation, and handwashing interventions are more effective at reducing diarrhoea than individual interventions; and to investigate whether the combination of water, sanitation, handwashing, and nutrition interventions reduces growth faltering more than each individual intervention. A companion trial in Kenya evaluated the same objectives.^9^

## Methods

### Study design

The WASH Benefits Bangladesh study was a cluster-randomised trial conducted in rural villages in Gazipur, Kishoreganj, Mymensingh, and Tangail districts of Bangladesh (appendix p 2). We grouped pregnant women who lived near enough to each other into a cluster to allow delivery of interventions by a single community promoter. We hypothesised that the interventions would improve the health of the index child in each household. Each measurement round lasted about 1 year and was balanced across treatment arms and geography to minimise seasonal or geographical confounding when comparing outcomes across groups. We chose areas with low groundwater iron and arsenic (because these affect chlorine demand) and where no major water, sanitation, or nutrition programmes were ongoing or planned by the government or large non-government organisations. The study design and rationale have been published previously.^10^

The latrine component of the sanitation intervention was a compound level intervention. The drinking water and handwashing interventions were household level interventions. The nutrition intervention was a child-specific intervention. We assessed the diarrhoea outcome among all children in the compound who were younger than 3 years at enrolment, which could underestimate the effect of interventions targeted only to index households (drinking water, and handwashing) or index children (nutrition). After the study results were unmasked, we analysed diarrhoea prevalence restricted to index children (ie, children directly targeted by each intervention).

The study protocol was approved by the Ethical Review Committee at The International Centre for Diarrhoeal Disease Research, Bangladesh (PR-11063), the Committee for the Protection of Human Subjects at the University of California, Berkeley (2011-09-3652), and the institutional review board at Stanford University (25863).

### Participants

Rural households in Bangladesh are usually organised into compounds where patrilineal families share a common courtyard and sometimes a pond, water source, and latrine. Research assistants visited compounds in candidate communities. If compound residents reported no iron taste in their drinking water nor iron staining of their water storage vessels,^11^ and if a woman reported being in the first two trimesters of pregnancy, research assistants recorded the global positioning system coordinates of her household. We reviewed maps of plotted households and made clusters of eight expectant women who lived close enough to each other for a single community promoter to readily walk to each compound. We used a 1 km buffer around each cluster to reduce the potential for spillover between clusters (median buffer distance 2·6 km [IQR 1·8–3·7]). Participants gave written informed consent before enrolment.

The in utero children of enrolled pregnant women (index children) were eligible for inclusion if their mother was planning to live in the study village for the next 2 years, regardless of where she gave birth. Only one pregnant woman was enrolled per compound, but if she gave birth to twins, both children were enrolled. Children who were younger than 3 years at enrolment and lived in the compound were included in diarrhoea measurements.

### Randomisation and masking

Clusters were randomly allocated to treatment using a random number generator by a coinvestigator at University of California, Berkeley (BFA). Each of the eight geographically adjacent clusters was block-randomised to the double-sized control arm or one of the six interventions (water; sanitation; handwashing; water, sanitation, and handwashing; nutrition; or water, sanitation, handwashing, and nutrition). Geographical matching ensured that arms were balanced across locations and time of measurement.

Interventions included distinct visible components so neither participants nor data collectors were masked to intervention assignment, although the data collection and intervention teams were different individuals. Two investigators (BFA and JBC) did independent, masked statistical analyses from raw datasets to generate final estimates, with the true group assignment variable replaced with a re-randomised uninformative assignment variable. The results were unmasked after all analyses were replicated.

### Procedures

We used the Integrated Behavioural Model for Water Sanitation and Hygiene to develop the interventions over 2 years of iterative testing and revision.^12^ This model addresses contextual, psychosocial, and technological factors at the societal, community, interpersonal, individual, and habitual levels.

Community promoters delivered the interventions. These promoters were women who had completed at least 8 years of formal education, lived within walking distance of an intervention cluster, and passed a written and oral examination. Promoters attended multiple training sessions, including quarterly refreshers. Training addressed technical intervention issues, active listening skills, and strategies for the development of collaborative solutions with study participants. Promoters were instructed to visit intervention households at least once weekly in the first 6 months, and then at least once every 2 weeks. Promoters who delivered more complex interventions received longer formal training (table 1).

**Table 1:** Training of community health promoters and content of home visits for the six intervention groups

	**Water**	**Sanitation**	**Handwashing**	**Nutrition**	**Water, sanitation, and handwashing**	**Water, sanitation, handwashing, and nutrition**
Training*						
Duration of initial training	4 days	4 days	4 days	5 days	5 days	9 days
Duration of refresher training	1 day	1 day	1 day	1 day	1 day	1 day
**Implementation**†						
Technology and supplies provided	Insulated storage container for drinking water; Aquatabs (Medentech, Ireland)	Sani-scoop; potty; double-pit pour flush improved latrine	Handwashing station; storage bottle for soapy water; laundry detergent sachets for preparation of soapy water	LNS (Nutriset, France); storage container for LNS	Same as individual water, sanitation, and handwashing interventions	Same as individual water, sanitation, handwashing, and nutrition interventions
Key behavioural recommendations delivered by promoters	Targeted children drink treated, safely stored water	Family use double pit latrines; potty train children; safely dispose of faeces into latrine or pit	Family wash hands with soap after defecation and during food preparation	Exclusive breastfeeding up to 180 days; introduce diverse complementary food at 6 months; feed LNS from 6–24 months	Same as individual water, sanitation, and handwashing interventions	Same as individual water, sanitation, handwashing, and nutrition interventions
Population targeted	Children younger than 5 years living in index households	Whole compound for latrines; index households for potty training and safe faeces disposal	Residents of index households	Index children (targeted through mother)	Same as individual water, sanitation, and handwashing interventions	Same as individual water, sanitation, handwashing, and nutrition interventions
Emphasis during visits after refresher training	Safe storage of water, children drink only treated and safely stored water	Latrine cleanliness; maintenance; pit switching	Handwashing before food preparation	Dietary diversity during complementary feeding; provide LNS even if child is unwell	Same as individual water, sanitation, and handwashing interventions	Same as individual water, sanitation, handwashing, and nutrition interventions

LNS=lipid-based nutrient supplement.

* Common across all arms: roles and responsibilities, introduction to behaviour change principles, and interpersonal and counselling communication skills. Specific for each intervention: technology installation and use, onsite demonstration of use in the home, resupplying and restocking, problem solving challenges to technology use, and adoption of behaviours. Refresher training was done 12–15 months after start of intervention; content was based on analysis of reasons for gap between goals for uptake and actual uptake and addressed reasons for low uptake (specific to each intervention).

† Promoter visits were intended to teach participants how to use technologies and how to use and restock products; arrange for social support; communicate benefits of use and practice and changes in social norms; congratulate and encourage; problem-solve as needed; and inspire. Techniques used included counselling via flipcharts and cue cards, onsite demonstrations of technologies and products, video dramas, storytelling, games, and songs. Promoter's guides detailed the visit objective, target audience, and the specific steps and materials to be used.

After the hardware was installed, household visits involved promoters greeting target household members, checking for the presence and functionality of hardware and signs of use, observing any of the recommended practices, and then following a structured plan for that visit. For each visit, a promoter's guide detailed the visit objective, the target audience, the specific steps, and materials to be used. Discussions, video dramas, storytelling, games, songs, and training on hardware maintenance were included in different visits. The breadth of the curriculum varied by the complexity of the intervention. Promoters delivering combined interventions were expected to spend sufficient time to cover all of the behavioural objectives with target households. Promoters did not visit control households. Promoters received a monthly stipend equivalent to US$20, comparable to the local compensation for 5 days of agricultural labour.

The water intervention, which was modelled on a successful intervention from a previous trial,^11^ provided a 10 L vessel with a lid, tap, and regular supply of sodium dichloroisocyanurate tablets (Medentech, Wexford, Ireland) to the household of index children. Households were encouraged to fill the vessel, add one 33 mg tablet, and wait 30 min before drinking the water. All household members, but especially children younger than 5 years, were encouraged to drink only chlorine-treated water. Non-index households in the compound did not receive the water intervention.

The latrine component of the sanitation intervention targeted all households in the compound. All latrines that did not have a slab, a functional water seal, or a construction that prevented surface runoff of a faecal stream into the community were replaced. If the index household did not have their own latrine, the project built one. The standard project intervention latrine was a double pit latrine with a water seal.^13^ Each pit had five concrete rings that were 0·3 m high. When the initial pit filled, the superstructure and slab could be moved to the second pit. In the less than 2% of cases where there was insufficient space for a second pit or the water table was too high for a pit that was 1·5 m deep, the design was adapted. Nearly all households (99%) provided labour and modest financial contributions towards the construction of the latrines. All households in sanitation intervention compounds also received a sani-scoop, which is a hand tool for the removal of faeces from the compound,^14^ and child potties if they had any children younger than 3 years.^15^ Promoters encouraged mothers to teach their children to use the potties, to safely dispose of faeces in latrines, and to regularly remove animal and human faeces from the compound.

The handwashing intervention targeted households with index children. These households received two handwashing stations, one with a 40 L water reservoir placed near the latrine and a 16 L reservoir for the kitchen. Each handwashing station included a basin to collect rinse water and a soapy water bottle.^16^ Promoters also provided a regular supply of detergent sachets for making soapy water. Promoters encouraged residents to wash their hands with soapy water before preparing food, before eating or feeding a child, after defecating, and after cleaning a child who has defected.

We aimed to deploy interventions so that index children were born into households with the interventions in place. In the combined intervention arms, the sanitation intervention was implemented first, followed by handwashing and then water treatment.

The nutrition intervention targeted index children. Promoters gave study mothers with children aged 6–24 months two 10 g sachets per day of lipid-based nutrient supplement (LNS; Nutriset; Malaunay, France) that could be mixed into the child's food. Each sachet provided 118 kcal, 9·6 g fat, 2·6 g protein, 12 vitamins, and ten minerals. Promoters explained that LNS should not replace breastfeeding or complementary foods and encouraged caregivers to exclusively breastfeed their children during the first 6 months and to provide a diverse, nutrient-dense diet using locally available foods for children older than 6 months. Intervention messages were adapted from the Alive & Thrive programme in Bangladesh.^17^

### Outcomes

Primary outcomes were caregiver-reported diarrhoea among all children who were in utero or younger than 3 years at enrolment in the past 7 days (based on all data from year 1 and year 2) and length-for-age *Z* score at year 2 in index children. Secondary outcomes included length-for-age *Z* score at year 1; weight-for-length *Z* score, weight-for-age *Z* score, head circumference-for-age *Z* score at year 1 and year 2; and prevalence of moderate stunting (length-for-age *Z* score less than −2), severe stunting (length-for-age *Z* score less than −3) underweight (weight-for-age *Z* score less than −2), and wasting (weight-for-age *Z* score less than −2). All-cause mortality among index children was a tertiary outcome.^10^ Full details on exclusion criteria, measurement protocols, and outcome definitions are in the appendix (p 21–27).

Outcome and adherence was assessed by a team of university graduates who were not involved in the delivery or promotion of interventions. They received a minimum of 21 days of formal training. The mother of the index child answered the interview questions.

We defined diarrhoea as at least three loose or watery stools within 24 h or at least one stool with blood.^18^ We assessed diarrhoea in the preceding 7 days among index children and among children who lived in enrolled compounds and who were younger than 3 years at enrolment and so would be expected to remain under 5 years of age throughout the trial. Diarrhoea was assessed at about 16 months and 28 months after enrolment. We included caregiver-reported bruising or abrasion as a negative control outcome.^19^

We calculated *Z* scores for length for age, weight for length, weight for age, and head circumference for age using the WHO 2006 child growth standards. Child mortality was assessed at the two follow-up evaluation visits based on caregiver interview. Length-for-age *Z* scores were measured at about 28 months after enrolment when index children would average about 24 months of age. Trained anthropometrists followed standard protocols^20^ and measured recumbent length (to 0·1 cm) and weight without clothing in duplicate; if the two values disagreed (>0·5 cm for length, 0·1 kg for weight) they repeated the measure until replicates fell within the error tolerance. We excluded children from *Z*-score analyses if their measurements were outside biologically plausible ranges according to WHO recommendations.^20^

### Statistical analyses

Sample size calculations for the two primary outcomes were based on a relative risk of diarrhoea of 0·7 or smaller (assuming a 7-day prevalence of 10% in the control group^21^) and a minimum detectable effect of 0·15 length-for-age *Z* score for comparisons of any intervention against control, accounting for repeated measures within clusters. The calculations assumed a type I error (α) of 0·05 and power (1–β) of 0·8, a one-sided test for a two-sample comparison of means, and 10% loss to follow-up. Sample size calculations indicated 90 clusters per group, each with eight children. Full details are given in appendix 4 of our study protocol.^10^

We analysed participants according to their randomised assignment (intention to treat), regardless of adherence to the intervention. Since randomisation was geographically pair-matched in blocks of eight clusters, we estimated unadjusted prevalence differences and ratios using a pooled Mantel-Haenszel estimator that stratified by matched pair.

We used paired *t* tests and cluster-level means for unadjusted *Z* score comparisons. For each comparison, we calculated two p values (two-sided): one for the test that mean differences were different from zero and a second to test for any difference between groups in the full distribution using permutation tests with the Wilcoxon signed-rank statistic. Secondary adjusted analyses controlled for prespecified, prognostic baseline covariates using data-adaptive, targeted maximum likelihood estimation. To assess whether interventions affected nearby clusters, we estimated the difference in primary outcomes between control compounds at different distances from intervention compounds. We did not adjust for multiple comparisons.^22^

Analyses were done in *R* (version 3.2.3). We tested for the presence of between-cluster spillover effects using a non-parametric method described in the prespecified analysis plan.

The trial is registered at ClinicalTrials.gov, number NCT01590095. The International Centre for Diarrhoeal Disease Research, Bangladesh convened a data and safety monitoring board and oversaw the study.

### Role of the funding source

The funders of the study approved the study design, but had no role in data collection, data analysis, data interpretation, or writing of the report. The corresponding author had full access to all data in the study and had final responsibility for the decision to submit for publication.

## Results

**Figure 1:**
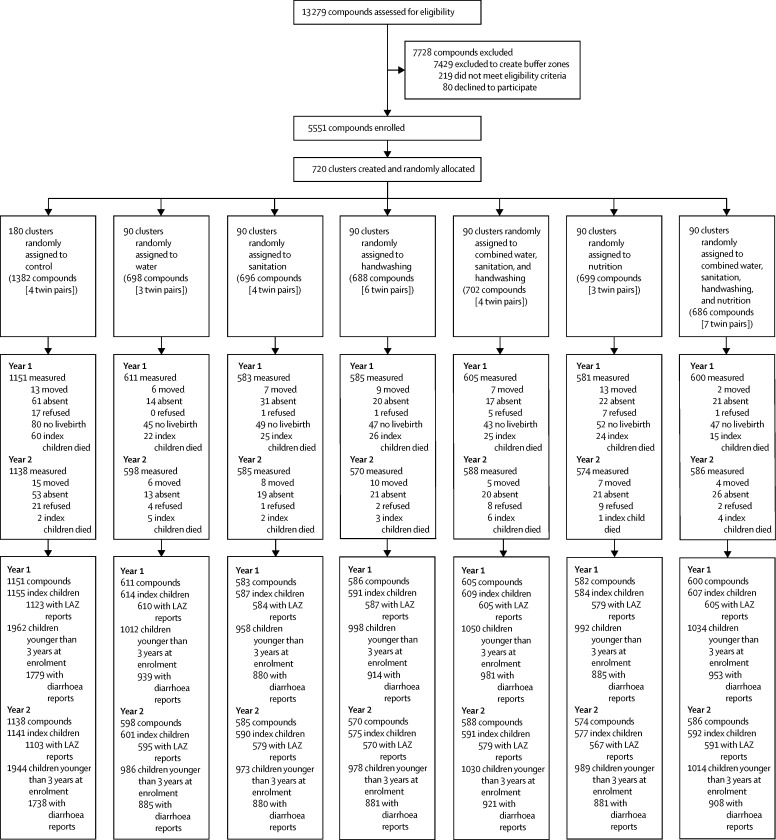
Trial profile and analysis populations for primary outcomes LAZ=length-for-age *Z* scores.

Fieldworkers identified 13 279 compounds with a pregnant woman in her first or second trimester; over half were excluded to create 1 km buffer zones between intervention areas. Between May 31, 2012, and July 7, 2013, we randomly allocated 720 clusters and enrolled 5551 pregnant women in 5551 compounds to an intervention or the control group (figure 1). Index children in 912 (16%) enrolled compounds did not complete follow-up, most commonly because they were not born alive (361 [7%]) or died before the final assessment (220 [4%]). 109 (2%) households moved, 175 (3%) were absent on repeated follow-up, and 47 (<1%) withdrew (figure 1). 4667 (93%) of 4999 surviving index children were measured at year 2, with length-for-age *Z* scores for 4584 (92%) children.

There were a median of two households (IQR 1–3, range 1–11) per compound. Most index households (4108 [74%] of 5551) collected drinking water from shallow tubewells. At enrolment, about half (2976 [54%] of 5551) of households owned their own latrine; most (4979 [90%] of 5551 households) used a latrine that had a concrete slab, and a quarter (1370 [25%] of 5551) had a functional water seal. Baseline characteristics of enrolled households were similar across groups (table 2).

**Table 2:** Baseline characteristics by intervention group

		**Control (n=1382)**	**Water treatment (n=698)**	**Sanitation (n=696)**	**Handwashing (n=688)**	**Water, sanitation, and handwashing (n=702)**	**Nutrition (n=699)**	**Water, sanitation, and handwashing, and nutrition (n=686)**
**Maternal**								
Age (years)		24 (5)	24 (5)	24 (5)	24 (5)	24 (5)	24 (5)	24 (6)
Years of education		6 (3)	6 (3)	6 (3)	6 (3)	6 (3)	6 (3)	6 (3)
**Paternal**								
Years of education		5 (4)	5 (4)	5 (4)	5 (4)	5 (4)	5 (4)	5 (4)
Works in agriculture		414 (30%)	224 (32%)	204 (29%)	249 (36%)	216 (31%)	232 (33%)	207 (30%)
**Household**								
Number of people		5 (2)	5 (2)	5 (2)	5 (2)	5 (2)	5 (2)	5 (2)
Has electricity		784 (57%)	422 (60%)	408 (59%)	405 (59%)	426 (61%)	409 (59%)	412 (60%)
Has a cement floor		145 (10%)	82 (12%)	85 (12%)	55 (8%)	77 (11%)	67 (10%)	72 (10%)
Acres of agricultural land owned		0·15 (0·21)	0·14 (0·20)	0·14 (0·22)	0·14 (0·20)	0·15 (0·23)	0·16 (0·27)	0·14 (0·38)
**Drinking water**								
Shallow tubewell is primary water source		1038 (75%)	500 (72%)	519 (75%)	482 (70%)	546 (78%)	519 (74%)	504 (73%)
Has stored water at home		666 (48%)	353 (51%)	341 (49%)	347 (50%)	304 (43%)	301 (43%)	331 (48%)
Reported treating water yesterday		4 (0%)	1 (0%)	0 (0%)	1 (0%)	0 (0%)	0 (0%)	2 (0%)
**Sanitation**								
Daily defecating in the open								
	Adult men	97 (7%)	39 (6%)	52 (8%)	64 (9%)	54 (8%)	59 (9%)	50 (7%)
	Adult women	62 (4%)	18 (3%)	33 (5%)	31 (5%)	29 (4%)	39 (6%)	24 (4%)
	Children aged 8 to <15 years	53 (10%)	25 (9%)	28 (9%)	43 (15%)	30 (10%)	23 (8%)	28 (10%)
	Children aged 3 to <8 years	267 (38%)	141 (37%)	137 (38%)	137 (39%)	137 (38%)	129 (39%)	134 (37%)
	Children aged 0 to <3 years	245 (82%)	112 (85%)	117 (84%)	120 (85%)	123 (79%)	128 (85%)	123 (88%)
Latrine								
	Owned*	750 (54%)	363 (52%)	374 (54%)	372 (54%)	373 (53%)	377 (54%)	367 (53%)
	Concrete slab	1251 (95%)	644 (95%)	610 (92%)	613 (93%)	620 (93%)	620 (94%)	621 (94%)
	Functional water seal	358 (31%)	183 (31%)	177 (30%)	162 (28%)	152 (26%)	183 (31%)	155 (27%)
	Visible stool on slab or floor	625 (48%)	350 (53%)	332 (52%)	335 (52%)	289 (44%)	331 (51%)	298 (46%)
	Owned a child potty	61 (4%)	27 (4%)	28 (4%)	35 (5%)	27 (4%)	36 (5%)	30 (4%)
Human faeces observed in the								
	House	114 (8%)	65 (9%)	56 (8%)	70 (10%)	48 (7%)	58 (8%)	49 (7%)
	Child's play area	21 (2%)	6 (1%)	6 (1%)	8 (1%)	7 (1%)	8 (1%)	7 (1%)
**Handwashing location**								
Within six steps of latrine								
	Has water	178 (14%)	83 (13%)	81 (13%)	63 (10%)	67 (10%)	62 (10%)	72 (11%)
	Has soap	88 (7%)	50 (8%)	48 (8%)	34 (5%)	42 (7%)	32 (5%)	36 (6%)
Within six steps of kitchen								
	Has water	118 (9%)	51 (8%)	51 (8%)	45 (7%)	61 (9%)	61 (9%)	60 (9%)
	Has soap	33 (3%)	18 (3%)	14 (2%)	13 (2%)	15 (2%)	23 (4%)	18 (3%)
**Nutrition**								
Household is food secure†		932 (67%)	495 (71%)	475 (68%)	475 (69%)	482 (69%)	479 (69%)	485 (71%)

Data are n (%) or mean (SD). Percentages were estimated from slightly smaller denominators than those shown at the top of the table for the following variables due to missing values: mother's age; father's education; father works in agriculture; acres of land owned; open defecation; latrine has a concrete slab; latrine has a functional water seal; visible stool on latrine slab or floor; ownership of child potty; observed faeces in the house or child's play area; and handwashing variables.

* Households in these communities who do not own a latrine typically share a latrine with extended family members who live in the same compound.

† Assessed by the Household Food Insecurity Access Scale.

Measures of intervention adherence included presence of stored drinking water with detectable free chlorine (>0·1 mg/L), a latrine with a functional water seal, presence of soap at the primary handwashing location, and reported consumption of LNS sachets. Intervention-specific adherence measures were all greater than 75% in households assigned to the relevant intervention and were substantially higher than practices in the control group. Adherence was similar in the single water, sanitation, handwashing, and nutrition intervention groups compared with the two groups that combined interventions (table 3). Adherence was similar at 1-year and 2-year follow-up.

**Table 3:** Measures of intervention adherence by study group at enrolment and at 1-year and 2-years follow-up

	**Control**	**Water**	**Sanitation**	**Handwashing**	**Washing, sanitation, and handwashing**	**Nutrition**	**Washing, sanitation, handwashing, and nutrition**
**Number of compounds assessed**							
Enrolment	1382 (100%)	698 (100%)	696 (100%)	688 (100%)	702 (100%)	699 (100%)	686 (100%)
Year 1	1151 (83%)	611 (88%)	583 (84%)	585 (85%)	605 (86%)	581 (83%)	600 (87%)
Year 2	1138 (82%)	598 (86%)	585 (84%)	570 (83%)	588 (84%)	574 (82%)	586 (85%)
**Stored drinking water**							
Enrolment	666 (48%)	353 (51%)	341 (49%)	347 (50%)	304 (43%)	301 (43%)	331 (48%)
Year 1	503 (44%)	587 (96%)	245 (42%)	266 (45%)	588 (97%)	229 (39%)	577 (96%)
Year 2	485 (43%)	567 (95%)	260 (44%)	267 (47%)	558 (95%)	225 (39%)	569 (97%)
**Stored drinking water has detectable free chlorine (>0·1 mg/L)**							
Enrolment	..	..	..	..	..	..	..
Year 1	..	467 (78%)	..	..	467 (79%)	..	472 (80%)
Year 2	..	488 (84%)	..	..	471 (81%)	..	501 (87%)
**Latrine with a functional water seal**							
Enrolment	358 (31%)	183 (31%)	177 (30%)	162 (28%)	152 (26%)	183 (31%)	155 (27%)
Year 1	308 (29%)	151 (27%)	554 (95%)	144 (27%)	573 (95%)	149 (28%)	564 (94%)
Year 2	324 (31%)	184 (33%)	568 (97%)	165 (32%)	567 (97%)	163 (31%)	561 (96%)
**No visible faeces on latrine slab or floor**							
Enrolment	625 (48%)	350 (53%)	332 (52%)	335 (52%)	289 (44%)	331 (51%)	298 (46%)
Year 1	658 (60%)	358 (61%)	516 (89%)	324 (58%)	522 (86%)	333 (60%)	527 (88%)
Year 2	612 (56%)	338 (58%)	502 (86%)	324 (60%)	484 (82%)	313 (58%)	495 (85%)
**Handwashing location has soap**							
Enrolment	294 (23%)	153 (24%)	155 (25%)	134 (22%)	155 (24%)	152 (24%)	149 (23%)
Year 1	283 (28%)	165 (30%)	158 (30%)	533 (91%)	546 (90%)	172 (34%)	536 (89%)
Year 2	320 (28%)	177 (30%)	180 (31%)	527 (92%)	531 (90%)	195 (34%)	540 (92%)
LNS sachets consumed (% expected)*							
Enrolment	..	..	..	..	..	..	..
Year 1	..	..	..	..	..	93%	94%
Year 2	..	..	..	..	..	94%	93%

Data are n (%) or %. Free chlorine in drinking water and LNS consumption were not measured at enrolment and were only measured in a subset of groups. LNS=lipid-based nutrient supplement.

* LNS adherence measured as proportion of 14 sachets consumed in the past week among index children ages 6–24 months (reported).

Diarrhoea prevalence in the control group was substantially below the 10% we had anticipated in our sample size calculations (table 4). Diarrhoea prevalence was particularly low during the first 9 months of observations, with evidence of seasonal epidemics in the control group during the monsoon seasons (appendix p 3).

**Table 4:** Diarrhoea prevalence 1 and 2 years (combined) after intervention

	**N**	Mean*prevalence	**Unadjusted**†**prevalence difference (95% CI)**	**Adjusted**‡**prevalence difference (95% CI)**
**Control *vs* intervention**				
Control	3517	5·7%	..	..
Water	1824	4·9%	−0·6 (−1·9 to 0·6)	−0·8 (−2·2 to 0·6)
Sanitation	1760	3·5%	−2·2 (−3·4 to −1·0)	−2·3 (−3·5 to −1·1)
Handwashing	1795	3·5%	−2·3 (−3·4 to −1·1)	−2·5 (−3·6 to −1·3)
Water, sanitation, and handwashing	1902	3·9%	−1·7 (−2·9 to −0·6)	−1·8 (−3·1 to −0·4)
Nutrition	1766	3·5%	−2·0 (−3·1 to −0·8)	−2·1 (−3·5 to −0·8)
Water, sanitation, handwashing, and nutrition	1861	3·5%	−2·2 (−3·3 to −1·0)	−2·2 (−3·4 to −1·0)
**Water, sanitation, and handwashing *vs* individual groups**				
Water, sanitation, and handwashing	1902	3·9%	..	..
Water	1824	4·9%	−1·2 (−2·5 to 0·2)	−0·9 (−2·2 to 0·5)
Sanitation	1760	3·5%	0·4 (−0·8 to 1·7)	0·5 (−0·8 to 1·8)
Handwashing	1795	3·5%	0·3 (−1·0 to 1·5)	0·7 (−0·6 to 1·9)

Among children younger than 3 years at enrolment.

* Post-intervention measurements in years 1 and 2 combined.

† Unadjusted estimates were estimated using a pair-matched Mantel-Haenszel analysis.

‡ Adjusted for prespecified covariates using targeted maximum likelihood estimation with data-adaptive model selection: field staff who collected data, month of measurement, household food insecurity, child age, child sex, mother's age, mothers height, mothers education level, number of children younger than 18 years in the household, number of individuals living in the compound, distance in minutes to the primary water source, household roof, floor, wall materials, and household assets.

**Figure 2:**
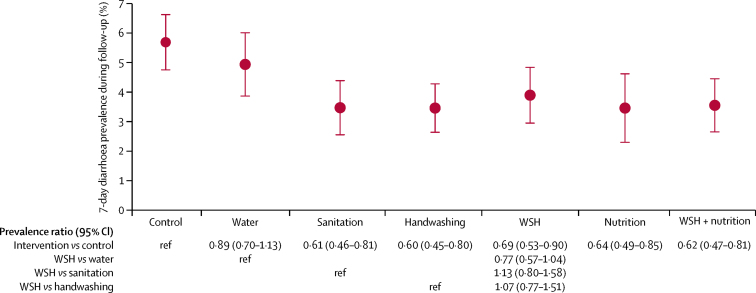
Intervention effects on diarrhoea prevalence in index children and children younger than 3 years at enrolment 1 and 2 years after intervention Data are mean (95% CI). ref=reference. WSH=water, sanitation, and handwashing.

Compared with the control group, index children and children who were younger than 3 years at enrolment and living in compounds where an index child received any intervention except water treatment had significantly decreased prevalence of diarrhoea at 1-year and 2-year follow-up (figure 2, table 4). The reductions in diarrhoea prevalence in the combined water, sanitation, and handwashing group were no larger than in the individual water, sanitation, or handwashing groups. Secondary adjusted analyses showed similar effect estimates of interventions on reported diarrhoea (table 4).

The effect of intervention was similar among the index children in targeted households (appendix p 10–11) compared with the analysis that included both index children and children younger than 3 years at enrolment who lived in the compound (figure 2); however, the point estimates of the prevalence ratio suggested that water or handwashing interventions did not have a notable effect on non-index children (appendix p 10–11).

There was no difference in prevalence of caregiver-reported bruising or abrasion between children in the control group and any of the intervention groups (appendix p 4).

**Figure 3:**
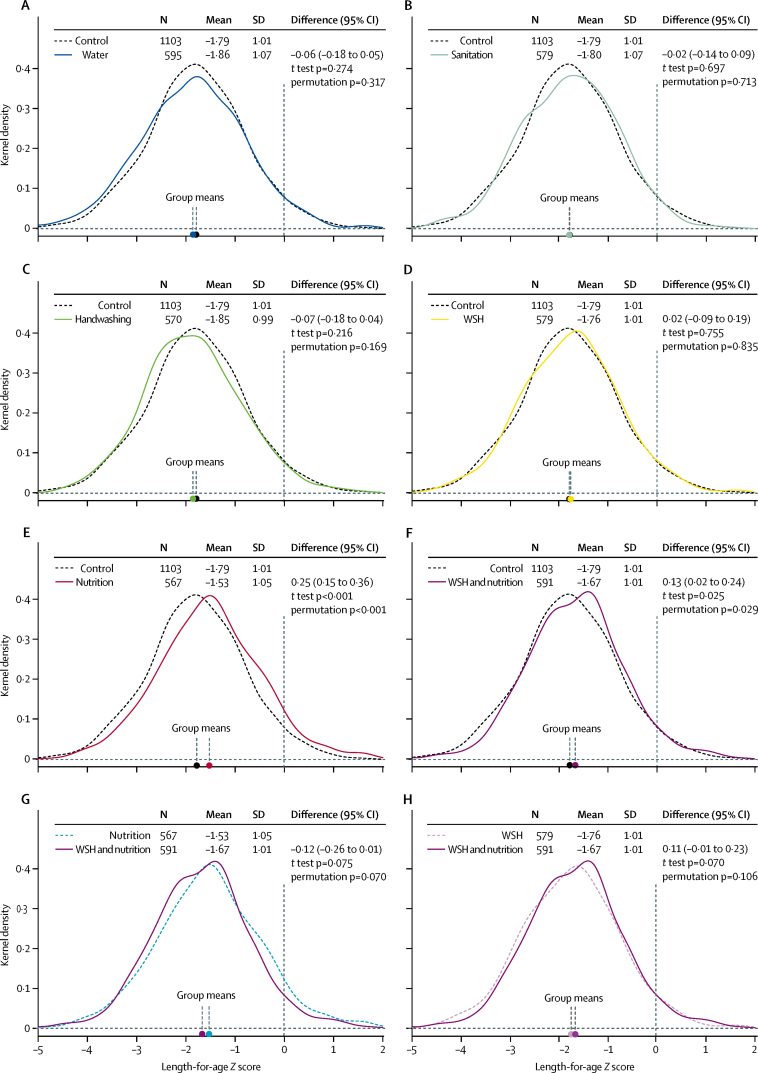
Intervention effects on length-for-age *Z* scores in 4584 children after 2 years of intervention Kernel density plots show the distribution of length-for-age *Z* scores among index children who were born into the study and were aged 18–28 months (median 22, IQR 21–24) at the time of measurement. Dashed lines are the comparison group distribution and solid lines are the active comparator distribution. (A) Water *vs* control. (B) Sanitation *vs* control. (C) Handwashing *vs* control. (D) WSH *vs* control. (E) Nutrition *vs* control. (F) WSH and nutrition *vs* control. (G) WSH and nutrition *vs* nutrition. (H) WSH and nutrition *vs* WSH. WSH=water, sanitation, and handwashing.

After 2 years of intervention (median age 22 months, IQR 21–24), mean length-for-age *Z* score in the control group was −1·79 (SD 1·01); children who received the nutrition intervention had an average increase of 0·25 (95% CI 0·15–0·36) in length-for-age *Z* scores; and children who received the water, sanitation, handwashing, and nutrition intervention had an average increase of 0·13 (0·02–0·24) in length-for-age *Z* scores (figure 3). After about 1 year of intervention (median age 9 months, IQR 8–10), children in the nutrition only group (but not children in the water, sanitation, handwashing, and nutrition group) were significantly taller than control children (appendix p 5).

Compared with control children, there was no significant difference in length-for-age *Z* scores in children receiving the water treatment (length-for-age *Z* score difference −0·06 [95% CI −0·18 to 0·05]), sanitation (−0·02 [–0·14 to 0·09]), handwashing (−0·07 [–0·18 to 0·04]), or water, sanitation, and handwashing interventions (0·02 [–0·09 to 0·13]; figure 3). Length-for-age *Z* scores were similar for children who received water, sanitation, handwashing, and nutrition and those who received nutrition only intervention (−0·12 [–0·26 to 0·01]).

After 2 years of intervention, children in the nutrition only or the water, sanitation, handwashing, and nutrition intervention had higher *Z* scores for length for age, weight for length, weight for age, and head circumference for age than did children in the control group (table 5). Children in the water treatment, sanitation, handwashing, or combined water, sanitation, and handwashing interventions had *Z* scores for length for age, weight for length, weight for age, and head circumference for age that were similar to controls (table 5).

**Table 5:** Child growth *Z* scores at 2-year follow-up

	**N**	**Mean (SD)**	**Difference *vs* control (95% CI)**	**Difference *vs* nutrition (95% CI)**	**Difference *vs* washing, sanitation, and handwashing (95% CI)**
**Weight-for-age *Z* score**					
Control	1121	−1·54 (1·00)	..	..	..
Water	599	−1·61 (1·04)	−0·07 (−0·19 to 0·04)	..	..
Sanitation	588	−1·52 (1·06)	−0·00 (−0·11 to 0·11)	..	..
Handwashing	573	−1·57 (1·00)	−0·04 (−0·16 to 0·08)	..	..
Water, sanitation, and handwashing	586	−1·53 (1·05)	0·00 (−0·09 to 0·10)	..	..
Nutrition	573	−1·29 (1·07)	0·24 (0·12 to 0·35)	..	..
Water, sanitation, handwashing, and nutrition	592	−1·42 (0·99)	0·13 (0·04 to 0·22)	−0·11 (−0·23 to 0·02)	0·12 (0·01 to 0·23)
**Weight-for-height *Z* score**					
Control	1104	−0·88 (0·93)	..	..	..
Water	596	−0·92 (0·97)	−0·04 (−0·14 to 0·05)	..	..
Sanitation	580	−0·85 (0·95)	0·01 (−0·09 to 0·11)	..	..
Handwashing	570	−0·86 (0·94)	0·00 (−0·11 to 0·12)	..	..
Water, sanitation, and handwashing	580	−0·88 (1·01)	0·00 (−0·10 to 0·11)	..	..
Nutrition	567	−0·71 (1·00)	0·15 (0·04 to 0·26)	..	..
Water, sanitation, handwashing, and nutrition	591	−0·79 (0·94)	0·09 (0·00 to 0·18)	−0·06 (−0·17 to 0·05)	0·09 (−0·03 to 0·21)
**Head circumference-for-age *Z* score**					
Control	1118	−1·61 (0·94)	..	..	..
Water	594	−1·63 (0·91)	−0·04 (−0·14 to 0·06)	..	..
Sanitation	584	−1·61 (0·86)	−0·01 (−0·10 to 0·09)	..	..
Handwashing	571	−1·56 (0·93)	0·05 (−0·06 to 0·15)	..	..
Water, sanitation, and handwashing	584	−1·59 (0·91)	0·03 (−0·07 to 0·12)	..	..
Nutrition	570	−1·45 (0·94)	0·16 (0·04 to 0·27)	..	..
Water, sanitation, handwashing, and nutrition	590	−1·51 (0·90)	0·11 (0·01 to 0·20)	−0·05 (−0·17 to 0·07)	0·08 (−0·04 to 0·19)

All three secondary outcomes were prespecified.

Compared with children living in control households, children enrolled in the nutrition only intervention were less likely to be stunted after 2 years; children enrolled in the water, sanitation, handwashing, and nutrition intervention were less likely to be severely stunted, or underweight (table 6). The proportion of children who were wasted was similar between the intervention and control groups.

**Table 6:** Prevalence of children stunted, severely stunted, wasted, and underweight at 2-year follow-up

	**n/N (%)**	**Difference *vs* control (95% CI)**	**Difference *vs* washing, sanitation, and handwashing (95% CI)**	**Difference *vs* nutrition (95% CI)**
Stunting*				
Control	451/1103 (41%)	..	..	..
Water	255/595 (43%)	2·4 (−2·6 to 7·3)	..	..
Sanitation	232/579 (40%)	−0·4 (−5·3 to 4·6)	..	..
Handwashing	263/570 (46%)	5·3 (0·2 to 10·3)	..	..
Water, sanitation, and handwashing	232/579 (40%)	−0·5 (−5·5 to 4·4)	..	..
Nutrition	186/567 (33%)	−7·7 (−12·4 to −2·9)	..	..
Water, sanitation, handwashing, and nutrition	221/591 (37%)	−3·8 (−8·6 to 1·1)	−2·8 (−8·4 to 2·8)	4·0 (−1·6 to 9·6)
**Severe stunting**†				
Control	124/1103 (11%)	..	..	..
Water	86/595 (15%)	3·3 (−0·1 to 6·7)	..	..
Sanitation	65/579 (11%)	0·1 (−3·0 to 3·3)	..	..
Handwashing	65/570 (11%)	0·2 (−3·0 to 3·4)	..	..
Water, sanitation, and handwashing	59/579 (10%)	−1·0 (−4·1 to 2·1)	..	..
Nutrition	47/567 (8%)	−2·8 (−5·7 to 0·2)	..	..
Water, sanitation, handwashing, and nutrition	50/591 (9%)	−3·0 (−5·9 to 0·0)	−1·9 (−5·2 to 1·4)	−0·3 (−3·5 to 3·0)
**Wasting**†				
Control	118/1104 (11%)	..	..	..
Water	73/596 (12%)	1·8 (−1·4 to 5·0)	..	..
Sanitation	65/580 (11%)	0·9 (−2·3 to 4·0)	..	..
Handwashing	60/570 (11%)	0·1 (−3·1 to 3·2)	..	..
Water, sanitation, and handwashing	69/580 (12%)	1·4 (−1·8 to 4·6)	..	..
Nutrition	50/567 (9%)	−1·6 (−4·5 to 1·3)	..	..
Water, sanitation, handwashing, and nutrition	52/591 (9%)	−1·7 (−4·7 to 1·2)	−2·8 (−6·3 to 0·7)	0·2 (−3·0 to 3·5)
**Underweight**†				
Control	344/1121 (31%)	..	..	..
Water	213/599 (36%)	5·3 (0·7 to 10·0)	..	..
Sanitation	179/588 (30%)	0·3 (−4·3 to 4·9)	..	..
Handwashing	197/573 (34%)	3·9 (−0·9 to 8·7)	..	..
Water, sanitation, and handwashing	192/586 (33%)	2·2 (−2·4 to 6·8)	..	..
Nutrition	149/573 (26%)	−4·2 (−8·6 to 0·3)	..	..
Water, sanitation, handwashing, and nutrition	148/592 (25%)	−5·8 (−10·2 to −1·4)	−7·8 (−12·9 to −2·6)	−1·7 (−6·6 to 3·3)

* Prespecified secondary outcome.

† Prespecified tertiary outcome.

Prespecified adjusted analyses found similar effect estimates on anthropometric outcomes with similar efficiency (appendix p 12–15). There was no evidence of between-cluster spillover effects (appendix p 8, 9 and 17–20).

In the control group, the cumulative incidence of child mortality was 4·7% (figure 1). Mortality in the individual water, sanitation, and handwashing groups and combined water, sanitation, and handwashing group was similar to controls. The two groups with a nutrition intervention had lower mortality: 3·8% for the nutrition group and 2·9% for the water, sanitation, handwashing, and nutrition group; this difference was significant for the combined group (risk difference water, sanitation, handwashing, and nutrition *vs* control −1·9% [95% CI −3·6 to −0·1]; p=0·0371; 38% relative reduction; appendix p 16).

## Discussion

In the WASH Benefits Bangladesh cluster-randomised controlled trial, the linear growth of children whose households had a chlorinated drinking water intervention, sanitation improvements, or handwashing intervention alone or in combination was no different than children in randomly assigned control households that received no intervention. Children in the nutrient supplement and counselling group grew somewhat taller than controls. Children in households that received a combination of water, sanitation, handwashing, and nutrition had no greater growth benefit than those receiving the nutrition-only intervention. Compared with control households, caregiver-reported diarrhoea prevalence was significantly decreased in households that received any of the interventions, except those who received only the drinking water treatment.

The trial's statistical power to detect small effects and high adherence to the interventions suggest that the absence of improvement in growth with water, sanitation, and handwashing interventions was a genuine null effect. These results suggest either that the hypothesis that exposure to faecal contamination contributes importantly to child growth faltering in Bangladesh is flawed or that the hypothesis remains valid but the water, sanitation, and handwashing interventions used in this trial did not reduce exposure to environmental pathogens sufficiently to reduce growth faltering. Future articles from our group will describe the effects of intervention on environmental contamination with faecal indicator bacteria and on the prevalence and concentration of enteric pathogens in stool specimens from children and thus provide insight on how effectively the interventions altered environmental contamination and enteropathogen transmission.

The effect of the nutrition intervention, which corrected one sixth of the growth deficit compared with international norms of healthy growth, was consistent with other randomised controlled trials of postnatal LNS that have reported variable and generally small effects on linear growth.^23^, ^24^, ^25^, ^26^, ^27^ This variation is probably because of contextual factors that affect a population's capacity to respond to an intervention. The water, sanitation, and handwashing intervention did not affect crucial contextual factors to amplify the effect of the nutrition interventions in rural Bangladesh. Continued research should explore interventions to reduce growth faltering.

Although intervention households generally reported less diarrhoea, people who received the intervention might have been grateful and, out of courtesy, reported less diarrhoea.^28^ However, compared with control households, intervention households reported no reduction in bruising or abrasions (negative control outcomes), so there was no evidence of systematic under-reporting of all health outcomes. It also seems unlikely that courtesy bias would affect each of the interventions except the drinking water intervention. The nutrition intervention might have led to improvements in breastfeeding practices or in essential fatty acids or micronutrient status, which could have contributed to improved gut epithelial immune response and thus less diarrhoea.^29^

The finding that drinking water treatment intervention had no notable effect on diarrhoea contrasts with our previous study of the identical intervention done between October, 2011, and November, 2012 in nearby communities that found a 36% reduction in reported diarrhoea.^11^ Restriction of the analysis to WASH Benefits index children who were targeted for the drinking water intervention led to a stronger treatment effect estimate (prevalence ratio 0·80 [95% CI 0·60–1·07]). Diarrhoea prevalence in the WASH Benefits control group (6%) was substantially lower than the 10% prevalence noted in a large prior study^21^ and the 11% prevalence in the control group of our previous study.^11^ Diarrhoeal prevalence characteristically varies substantially in nearby locations and from year to year.^30^ Diarrhoea prevalence in the control group of this WASH Benefits trial in rural Bangladesh was similar to diarrhoea prevalence among cohorts of children aged 1–4 years in the USA.^31^ At the time of the study, rotavirus immunisation had not been introduced into the Bangladesh national immunisation programme. The unexpectedly low diarrhoea prevalence among control children suggests decreased transmission of diarrhoea-causing pathogens during the WASH Benefits trial compared with recent evaluations. This low transmission provided less opportunity to interrupt transmission and less statistical power to show that interruption.

Combining interventions to improve drinking water quality, sanitation, and handwashing provided no additive benefit for the reduction of diarrhoea over single interventions. The unexpectedly low diarrhoea prevalence suggests low transmission of enteric pathogens through some of the pathways, which might have prevented any additive benefit from the combined interventions. Combined interventions did not compromise observed adherence to recommended practices. If a substantial proportion of the reduced diarrhoea was because of courtesy bias, this bias might mask subtle additive benefits. The only previous randomised controlled evaluations of multiple interventions versus single interventions also found no additive benefit of multiple components of water, sanitation, and handwashing on reported diarrhoea among children younger than 5 years.^7^, ^32^, ^33^ Because transmission pathways of enteropathogens vary by time and location, this absence of an additive effect with combined interventions is unlikely to generalise to all locations. However, these findings suggest that focusing resources on a single low-cost high-uptake intervention to a larger population might reduce diarrhoea prevalence more than would similar spending on more comprehensive approaches to smaller populations.

Children who received both the nutrition and the combined water, sanitation, and handwashing intervention were 38% less likely to die than children in the control group. Mortality was not a primary study outcome. Although the confidence limits are broad and the p value is borderline (p=0·037), a causal relationship from the interventions is plausible, since diarrhoea and poor nutrition are risk factors for death among young children in this setting. Notably, reduced mortality was only seen in the intervention groups that saw improved growth (nutrition groups), which were the groups with objective indicators of biological effect. Forthcoming investigations of the timing and causes of death assessed by verbal autopsy, distribution of enteropathogens among intervention groups, and effect of interventions on respiratory disease will provide additional evidence to assess the biological plausibility of a causal relationship between the combined water, sanitation, handwashing, and nutrition intervention and reduced mortality.

The randomised design, balanced groups, and high adherence suggests that the absence of an association between water, sanitation, and handwashing interventions and growth is internally valid, but this intervention was implemented in one socioecological zone (rural Bangladesh) during a time of low diarrhoea prevalence. Reducing faecal exposure through household water, sanitation, and handwashing interventions might affect growth in settings with a different prevalence of gastrointestinal disease or mix of pathogens.^34^ Notably, water, sanitation, and handwashing interventions did not prevent growth faltering in this context where stunting is a prevalent public health issue and where adherence to the interventions was substantially higher than in typical programmatic interventions.^21^, ^35^, ^36^

The objective measures of uptake reflected the availability of infrastructure and supplies, but might over-represent actual use. Future articles from our group will include structured observation and other measures of uptake. Although more intensive interventions could lead to even better practices, it seems unlikely that large-scale routine programmes could implement interventions with such intensity.

Because the sanitation intervention targeted compounds with pregnant women, these interventions only reached about 10% of residents in villages where interventions were implemented. If a higher threshold of sanitation coverage is necessary to achieve herd protection, then this study design would preclude the detection of this effect. We used compounds as the unit of intervention because they enabled us to deliver intensive interventions with high adherence for thousands of newborn children. In addition, we expected compound-level faecal contamination to represent the dominant source of exposure for index children because of the physical separation of compounds, and because children younger than 2 years of age in these communities spent nearly all of their time in their own compound.

The combined water, sanitation, handwashing, and nutrition intervention had sustained high levels of adherence. Although the full range of benefits of these successfully integrated interventions are yet to be fully elucidated, our findings suggest there might be a survival benefit. Forthcoming articles by our group will report the effects of intervention on biomarkers of environmental enteric dysfunction, soil-transmitted helminth infection, enteric pathogen infection, biomarkers of inflammation and allostatic load, anaemia and nutritional biomarkers, and child language, motor development, and social skills.
