# Supplementary material for: Effects of water quality, sanitation, handwashing, and nutritional interventions on diarrhoea and child growth in rural Bangladesh: a cluster randomised controlled trial
Source: Lancet Glob Health. 2018 Jan 29;6(3):e302–15. doi: 10.1016/S2214-109X(17)30490-4 (PMC5809718; doi:10.1016/S2214-109X(17)30490-4)
Supplement: Supplementary appendix [file mmc1.pdf]

THE LANCET
Global Health

## Supplementary appendix

This appendix formed part of the original submission and has been peer reviewed. We post it as supplied by the authors.

Supplement to: Luby SL, Rahman M, Arnold BF, et al. Effects of water quality, sanitation, handwashing, and nutritional interventions on diarrhoea and child growth in rural Bangladesh: a cluster randomised controlled trial. *Lancet Glob Health* 2018; published online Jan 29. http://dx.doi.org/10.1016/S2214-109X(17)30490-4.

**Supplementary Information for**
# Effects of water quality, sanitation, handwashing, and nutritional interventions on diarrhoea and child growth in rural Bangladesh: a cluster randomised trial

## Table of Contents

Fig. S1: Location of study clusters ........ 2

Fig. S2 : Diarrhoea prevalence among children <36 months at enrollment by calendar month........ 3

Fig. S3 : Intervention effects on a negative control outcome of reported bruising or abrasions among children <36 months at enrollment. ........ 4

Fig. S4: Intervention effects on length for age Z-scores (LAZ) among 4,694 children after 1 year of intervention. ........ 5

Fig. S5 : Intervention effects on stunting prevalence among 4,584 children in the birth cohort after 2 years of intervention. ........ 7

Fig. S6: Plots of length-for-age Z-score by distance from each control compound to the nearest treated compound by arm ........ 8

Table S1: Diarrhea prevalence and unadjusted prevalence differences among index children and other children in the compound: 1 and 2-Year follow-up combined ........ 10

Table S2: Diarrhea prevalence and unadjusted prevalence ratios among index children and other children in the compound: 1 and 2-Year follow-up combined ........ 11

Table S3: Child growth Z-scores after 2 years of intervention – single and combined intervention arms minus control arm – Unadjusted and adjusted analyses ........ 12

Table S4: Child growth Z-scores after 2 years of intervention – Nutrition + WSH arm minus Nutrition and WSH arms – Unadjusted and adjusted analyses ........ 13

Table S5: Proportion of children stunted, severely stunted, wasted and underweight after 2 years of intervention – single and combined intervention arms minus control arm unadjusted and adjusted analyses ........ 14

Table S6: Proportion of children stunted, severely stunted, wasted and underweight after 2 years of intervention – Nutrition + WSH arm minus Nutrition and WSH arms unadjusted and adjusted analyses ........ 15

Table S7: All-cause mortality among index children 2 years after intervention ........ 16

Table S8: Between-cluster spillovers conditional on distance to the nearest treated compound - Unadjusted permutation test p-values ........ 17

Table S9: Between-cluster spillovers conditional on distance to the nearest treated compound - Adjusted permutation test p-values ........ 18

Table S10: Between-cluster spillovers on length-for-age z-score through shared institutions - Unadjusted permutation test p-values ........ 19

Table S11: Between-cluster spillovers on length-for-age z-score through shared institutions - Adjusted permutation test p-values ........ 20

Inclusion / Exclusion criteria—WASH Benefits Bangladesh protocol excerpt ........ 21

Outcome definitions – WASH Benefits Bangladesh excerpt ........ 22

Measurement protocols—WASH Benefits Bangladesh protocol excerpt ........ 23

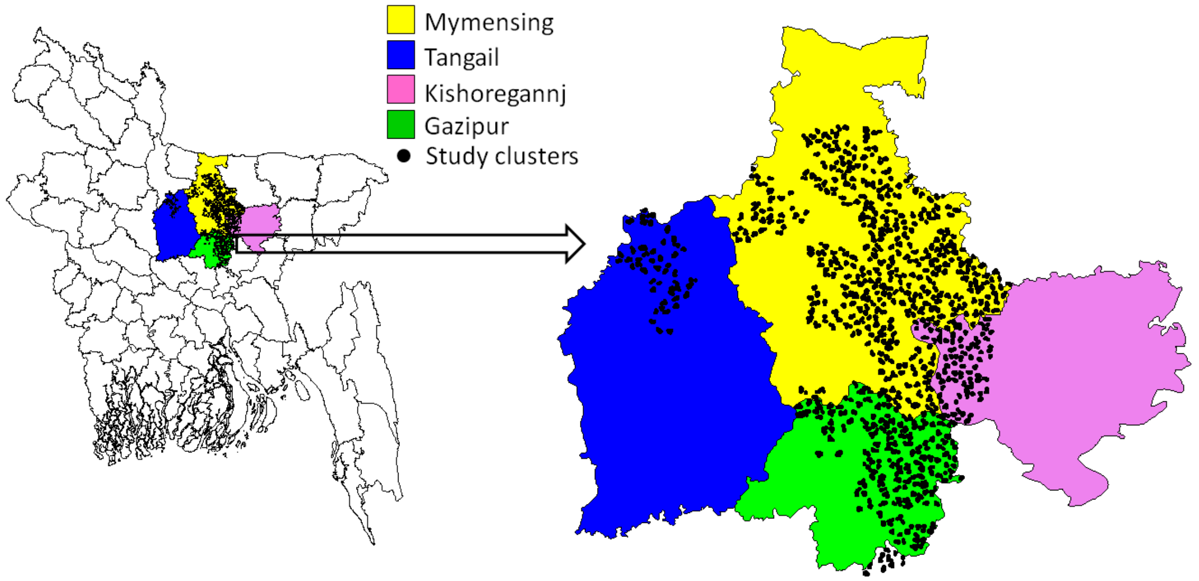


Fig. S1: Location of study clusters

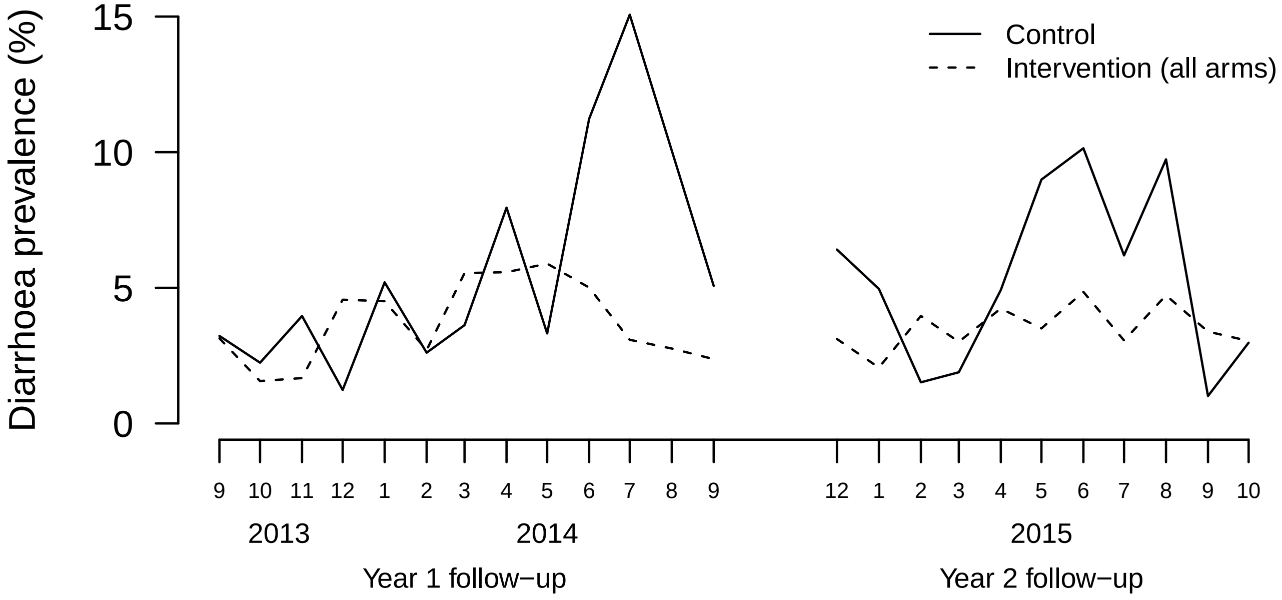


Fig. S2 : Diarrhoea prevalence among children <36 months at enrollment by calendar month. Individual children were measured only once at each round of follow-up, but due to the large study size each follow-up measurement spanned approximately one calendar year. Control and intervention clusters were geographically matched and matched clusters were measured concurrently. The seasonal monsoon in Bangladesh typically spans May to September. All intervention arms had similar prevalence during follow-up (Fig 3), and were combined into a single data series in this figure to have sufficient observations to estimate monthly prevalence. The control data series includes on average 145 observations per month (range: 73, 244) and the intervention data series includes on average 455 observations per month (range: 227, 732).

| Prevalence Ratio (95% CI) | Control | Water | Sanitation | Handwashing | Combined WSH | Nutrition | Combined Nutrion+WSH |
|---|---|---|---|---|---|---|---|
| Intervention v. Control | ref | 0.76 (0.53, 1.09) | 1.00 (0.71, 1.41) | 0.99 (0.70, 1.39) | 1.28 (0.95, 1.73) | 0.81 (0.56, 1.17) | 1.13 (0.82, 1.55) |
| WSH v. Water | | ref | | | 1.69 (1.15, 2.51) | | |
| WSH v. Sanitation | | | ref | | 1.29 (0.89, 1.86) | | |
| WSH v. Handwashing | | | | ref | 1.31 (0.92, 1.88) | | |

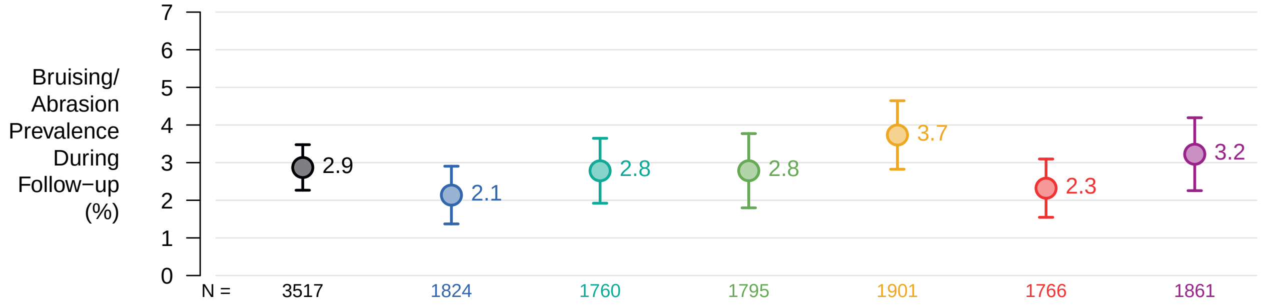


Fig. S3 : Intervention effects on a negative control outcome of reported bruising or abrasions among children <36 months at enrollment. Reported bruising or abrasions was measured twice during the study alongside diarrhoea symptoms, at 1 and 2 years after the delivery of interventions. Mantel-Haenszel prevalence ratios (stratified by matched pair) and their 95% confidence intervals (CI) compare each Intervention arm against the control arm, as well as the combined water, sanitation, and handwashing (WSH) package against single water, sanitation, or handwashing interventions.

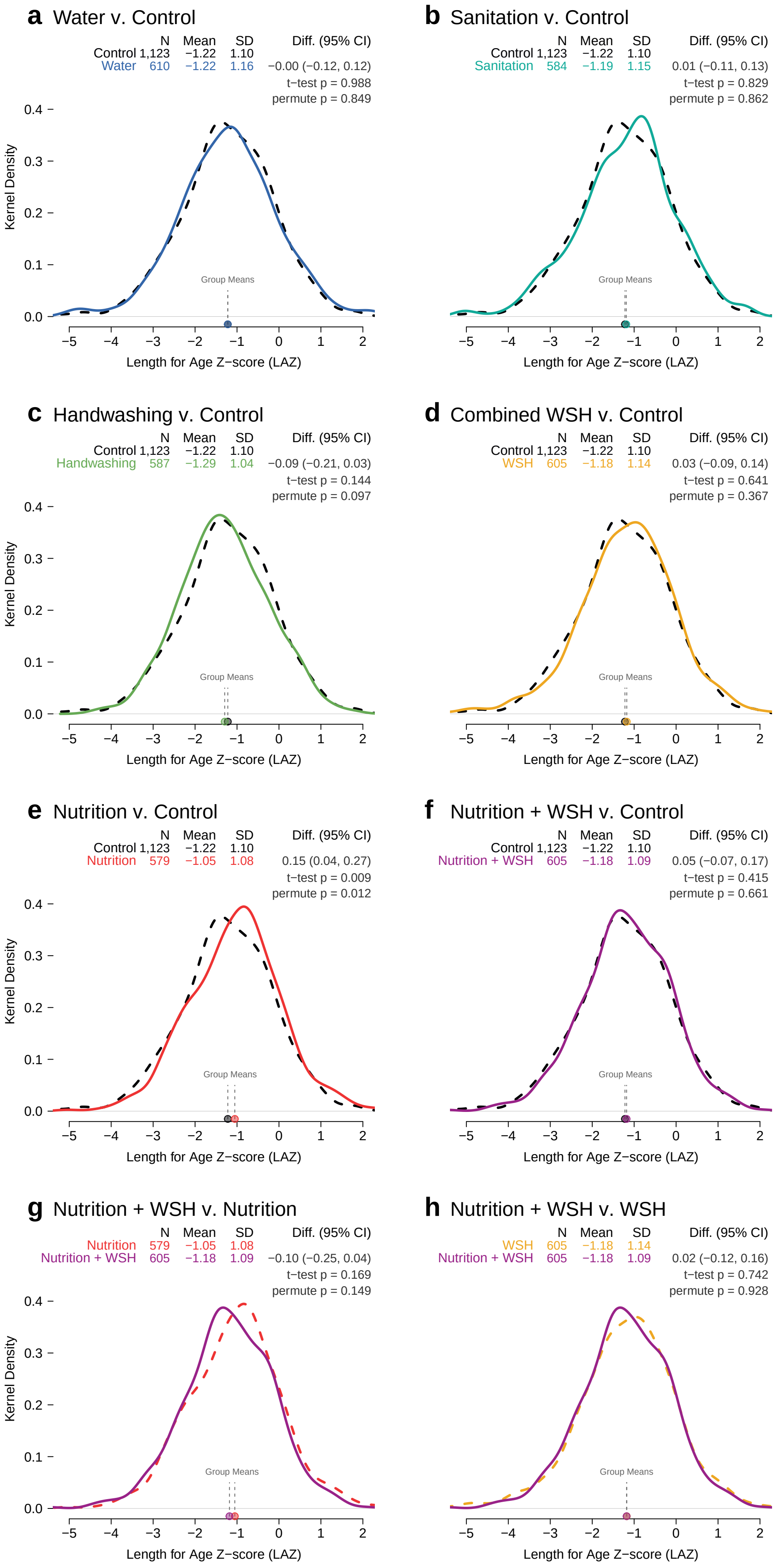


Fig. S4 : Caption on the next page.

Fig. S4: Intervention effects on length for age Z-scores (LAZ) among 4,694 children after 1 year of intervention. Kernel density plots summarize the distribution of LAZ among index children who were born into the study and were between 5-12 months (median = 9 months) at the time of measurement. In each panel, a dashed line illustrates the comparison group distribution and a solid line illustrates the active comparator distribution. **a-f**, each active intervention arm compared against the double-sized control arm; **g,h**, combined water, sanitation, handwashing (WSH) plus nutrition compared against either nutrition alone (**g**) or WSH alone (**h**). t-test p-values test whether differences in group means are different from zero; permutation p-values test the strong null hypothesis of no difference between groups using a Wilcoxon signed-rank test statistic.

| Difference (95% CI) | Control | Water | Sanitation | Handwashing | Combined WSH | Nutrition | Combined Nutrion+WSH |
|---|---|---|---|---|---|---|---|
| Intervention v. Control | ref | 2.4 (−2.6, 7.3) | −0.4 (−5.3, 4.6) | 5.3 (0.2, 10.3) | −0.5 (−5.5, 4.4) | −7.7 (−12.4, −2.9) | −3.8 (−8.6, 1.1) |
| Nutrition + WSH v. WSH | | | | | ref | | −2.8 (−8.4, 2.8) |
| Nutrition + WSH v. Nutrition | | | | | | ref | 4.0 (−1.6, 9.6) |

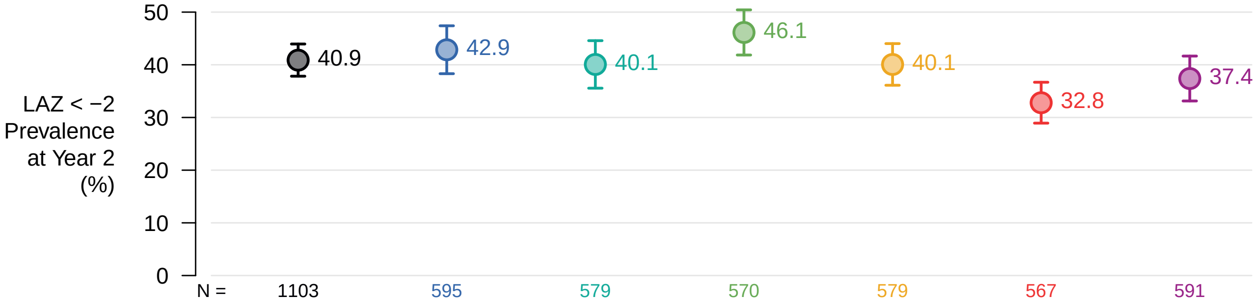


Fig. S5 : Intervention effects on stunting prevalence among 4,584 children in the birth cohort after 2 years of intervention. Children were between 18-28 months (median = 22 months) at the time of measurement. Mantel-Haenszel prevalence differences (stratified by matched pair) and their 95% confidence intervals (CI) compare each Intervention arm against the control arm, as well as the combined water, sanitation, handwashing (WSH) and nutrition package against combined WSH or nutrition interventions.

## Fig. S6: Plots of length-for-age Z-score by distance from each control compound to the nearest treated compound by arm

(black points are raw data; blue line is LOESS smoother of length-for-age Z-score mean)

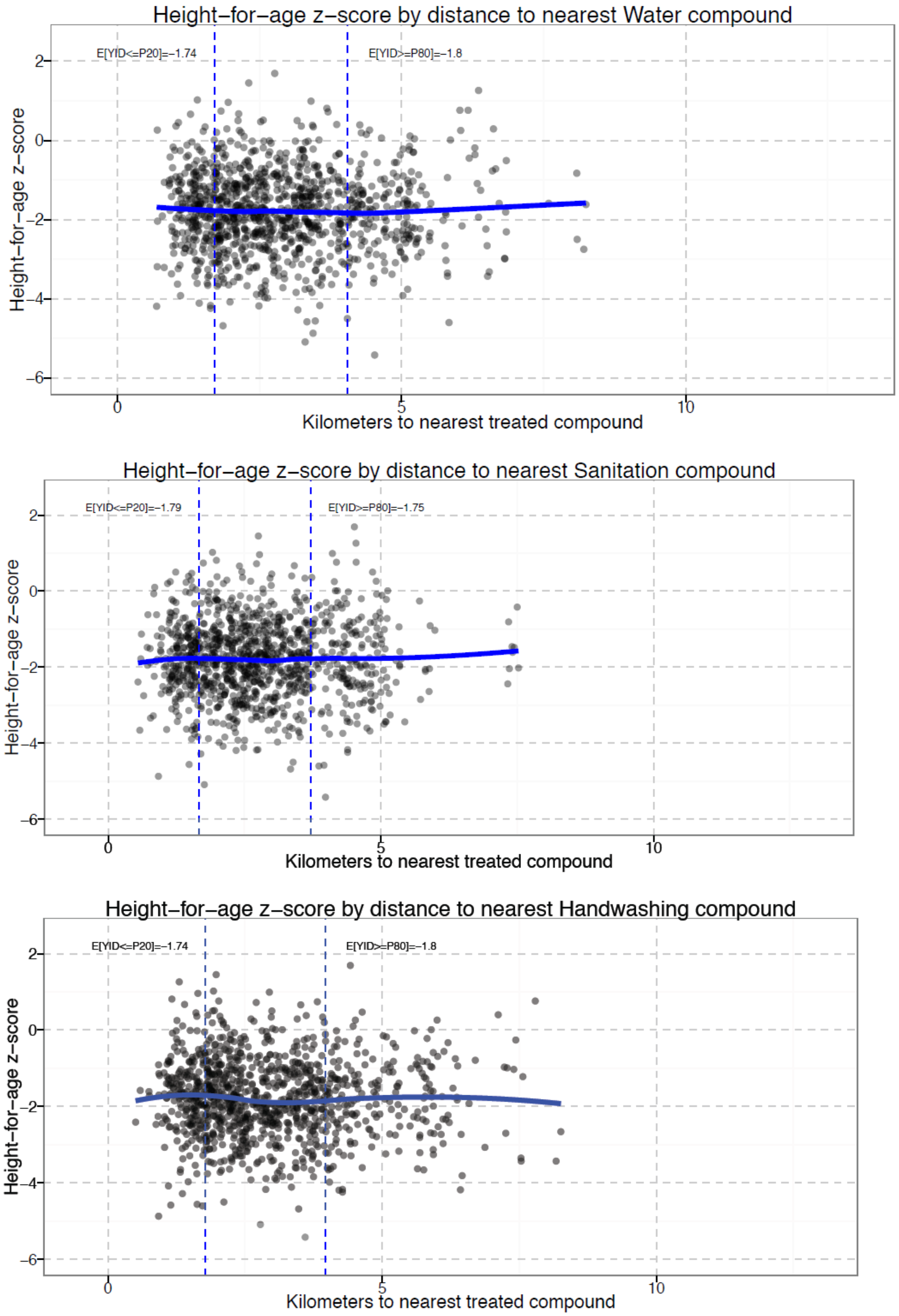

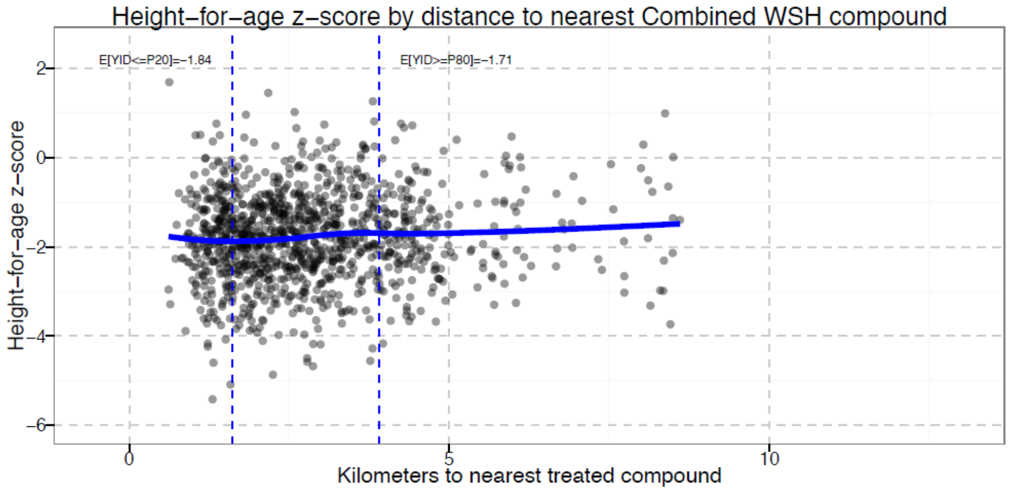
Height–for–age z–score by distance to nearest Combined WSH compound
E[Y|D<=P20]=–1.84
E[Y|D>=P80]=–1.71
Height–for–age z–score
Kilometers to nearest treated compound

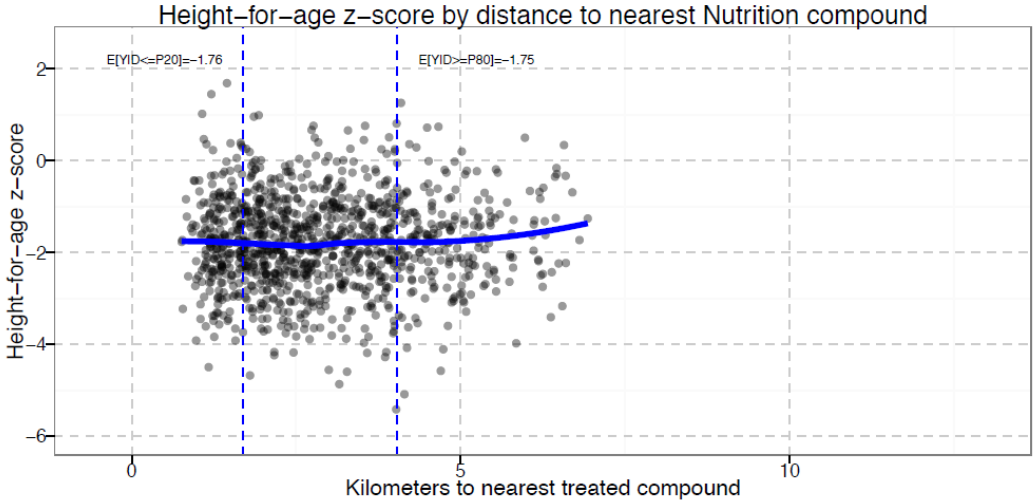
Height–for–age z–score by distance to nearest Nutrition compound
E[Y|D<=P20]=–1.76
E[Y|D>=P80]=–1.75
Height–for–age z–score
Kilometers to nearest treated compound

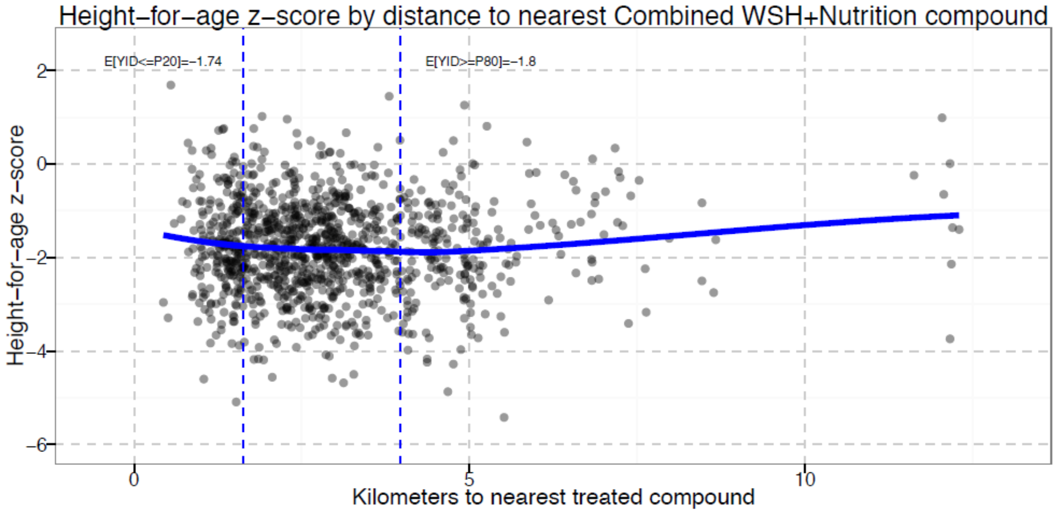
Height–for–age z–score by distance to nearest Combined WSH+Nutrition compound
E[Y|D<=P20]=–1.74
E[Y|D>=P80]=–1.8
Height–for–age z–score
Kilometers to nearest treated compound

## Table S1: Diarrhea prevalence and unadjusted prevalence differences among index children and other children in the compound: 1 and 2-Year follow-up combined

| | Index children | | | | Other children | | | |
|---|---|---|---|---|---|---|---|---|
| **Arm** | **N** | **n** | **Prevalence** | **PD* (95% CI)** | **N** | **n** | **Prevalence** | **PD (95% CI)** |
| Control | 2288 | 147 | 6.4 | -- | 1229 | 53 | 4.3 | -- |
| Water | 1208 | 61 | 5.0 | -1.3 (-2.8, 0.3) | 616 | 29 | 4.7 | 0.4 (-1.8, 2.6) |
| Sanitation | 1176 | 48 | 4.1 | -2.4 (-3.9, -0.9) | 584 | 13 | 2.2 | -1.9 (-3.7, -0.1) |
| Handwashing | 1162 | 32 | 2.8 | -3.6 (-5.0, -2.2) | 633 | 30 | 4.7 | 0.1 (-2.0, 2.3) |
| WSH | 1194 | 54 | 4.5 | -1.8 (-3.4, -0.3) | 708 | 20 | 2.8 | -1.9 (-3.6, -0.1) |
| Nutrition | 1159 | 46 | 4.0 | -2.2 (-3.7, -0.7) | 607 | 15 | 2.5 | -1.5 (-3.3, 0.4) |
| Nutrition + WSH | 1197 | 49 | 4.1 | -2.3 (-3.8, -0.8) | 664 | 17 | 2.6 | -2.6 (-4.3, -0.8) |

*Unadjusted prevalence difference estimated using a pair-matched Mantel-Haenszel analysis.

## Table S2: Diarrhea prevalence and unadjusted prevalence ratios among index children and other children in the compound: 1 and 2-Year follow-up combined

| | Index children | | | | Other children | | | |
|---|---|---|---|---|---|---|---|---|
| **Arm** | **N** | **n** | **Prevalence** | **PR* (95% CI)** | **N** | **n** | **Prevalence** | **PR (95% CI)** |
| Control | 2288 | 147 | 6.4 | -- | 1229 | 53 | 4.3 | -- |
| Water | 1208 | 61 | 5.0 | 0.80 (0.60, 1.07) | 616 | 29 | 4.7 | 1.09 (0.70, 1.71) |
| Sanitation | 1176 | 48 | 4.1 | 0.63 (0.46, 0.87) | 584 | 13 | 2.2 | 0.55 (0.29, 1.03) |
| Handwashing | 1162 | 32 | 2.8 | 0.43 (0.30, 0.63) | 633 | 30 | 4.7 | 1.03 (0.67, 1.60) |
| WSH | 1194 | 54 | 4.5 | 0.71 (0.52, 0.96) | 708 | 20 | 2.8 | 0.59 (0.34, 1.01) |
| Nutrition | 1159 | 46 | 4.0 | 0.65 (0.47, 0.90) | 607 | 15 | 2.5 | 0.64 (0.35, 1.17) |
| Nutrition + WSH | 1197 | 49 | 4.1 | 0.64 (0.47, 0.87) | 664 | 17 | 2.6 | 0.46 (0.25, 0.83) |

*Unadjusted prevalence ratio estimated using a pair-matched Mantel-Haenszel analysis.

## Table S3: Child growth Z-scores after 2 years of intervention – single and combined intervention arms minus control arm – Unadjusted and adjusted analyses

| | | | Difference from control (95% CI) | |
|---|---|---|---|---|
| **Outcome, Arm** | **N** | **Mean** | **Unadjusted** | **Adjusted*** |
| **Length-for-age Z-score** | | | | |
| Outcome, Arm | | | | |
| Control | 1103 | -1.79 | | |
| Water | 595 | -1.86 | -0.06 (-0.18, 0.05) | -0.04 (-0.14, 0.05) |
| Sanitation | 579 | -1.8 | -0.02 (-0.14, 0.09) | 0.03 (-0.07, 0.13) |
| Handwashing | 570 | -1.85 | -0.07 (-0.18, 0.04) | -0.04 (-0.13, 0.05) |
| Water + Sanitation + Handwashing (WSH) | 579 | -1.76 | 0.02 (-0.09, 0.13) | 0.05 (-0.04, 0.14) |
| Nutrition | 567 | -1.53 | 0.25 (0.15, 0.36) | 0.28 (0.18, 0.37) |
| Nutrition + WSH | 591 | -1.67 | 0.13 (0.02, 0.24) | 0.18 (0.09, 0.26) |
| **Weight-for-age Z-score** | | | | |
| Control | 1121 | -1.54 | | |
| Water | 599 | -1.61 | -0.07 (-0.19, 0.04) | -0.06 (-0.16, 0.03) |
| Sanitation | 588 | -1.52 | -0.00 (-0.11, 0.11) | 0.04 (-0.06, 0.14) |
| Handwashing | 573 | -1.57 | -0.04 (-0.16, 0.08) | 0.00 (-0.11, 0.11) |
| Water + Sanitation + Handwashing (WSH) | 586 | -1.53 | 0.00 (-0.09, 0.10) | 0.02 (-0.06, 0.10) |
| Nutrition | 573 | -1.29 | 0.24 (0.12, 0.35) | 0.26 (0.16, 0.36) |
| Nutrition + WSH | 592 | -1.42 | 0.13 (0.04, 0.22) | 0.16 (0.07, 0.24) |
| **Weight-for-height Z-score** | | | | |
| Control | 1104 | -0.88 | | |
| Water | 596 | -0.92 | -0.04 (-0.14, 0.05) | -0.04 (-0.13, 0.05) |
| Sanitation | 580 | -0.85 | 0.01 (-0.09, 0.11) | 0.04 (-0.06, 0.13) |
| Handwashing | 570 | -0.86 | 0.00 (-0.11, 0.12) | 0.02 (-0.09, 0.13) |
| Water + Sanitation + Handwashing (WSH) | 580 | -0.88 | 0.00 (-0.10, 0.11) | -0.01 (-0.11, 0.08) |
| Nutrition | 567 | -0.71 | 0.15 (0.04, 0.26) | 0.15 (0.05, 0.26) |
| Nutrition + WSH | 591 | -0.79 | 0.09 (0.00, 0.18) | 0.09 (-0.00, 0.18) |
| **Head circumference-for-age Z-score** | | | | |
| Control | 1118 | -1.61 | | |
| Water | 594 | -1.63 | -0.04 (-0.14, 0.06) | -0.02 (-0.11, 0.08) |
| Sanitation | 584 | -1.61 | -0.01 (-0.10, 0.09) | 0.00 (-0.09, 0.10) |
| Handwashing | 571 | -1.56 | 0.05 (-0.06, 0.15) | 0.06 (-0.04, 0.16) |
| Water + Sanitation + Handwashing (WSH) | 584 | -1.59 | 0.03 (-0.07, 0.12) | 0.01 (-0.07, 0.10) |
| Nutrition | 570 | -1.45 | 0.16 (0.04, 0.27) | 0.16 (0.06, 0.26) |
| Nutrition + WSH | 590 | -1.51 | 0.11 (0.01, 0.20) | 0.12 (0.02, 0.21) |

*Adjusted for pre-specified covariates using targeted maximum likelihood estimation with data-adaptive model selection [Arnold 2013, Balzer 2016]: Field staff who collected data, month of measurement, household food insecurity, child age, child sex, mother's age, mothers height, mothers education level, number of children < 18 years in the household, number of individuals living in the compound, distance in minutes to the primary water source, household roof, floor, wall materials, household assets.

## Table S4: Child growth Z-scores after 2 years of intervention – Nutrition + WSH arm minus Nutrition and WSH arms – Unadjusted and adjusted analyses

| | | | Nutrition + WSH minus single arms (95% CI) | |
|---|---|---|---|---|
| **Outcome, Arm** | **N** | **Mean** | **Unadjusted** | **Adjusted*** |
| **Length-for-age Z-score** | | | | |
| Nutrition + WSH | 591 | -1.67 | | |
| Nutrition | 567 | -1.53 | -0.12 (-0.26, 0.01) | -0.11 (-0.23, 0.00) |
| WSH | 579 | -1.76 | 0.11 (-0.01, 0.23) | 0.11 (0.02, 0.21) |
| **Weight-for-age Z-score** | | | | |
| Nutrition + WSH | 592 | -1.42 | | |
| Nutrition | 573 | -1.29 | -0.11 (-0.23, 0.02) | -0.11 (-0.22, -0.01) |
| WSH | 586 | -1.53 | 0.12 (0.01, 0.23) | 0.13 (0.03, 0.22) |
| **Weight-for-height Z-score** | | | | |
| Nutrition + WSH | 591 | -0.79 | | |
| Nutrition | 567 | -0.71 | -0.06 (-0.17, 0.05) | -0.07 (-0.17, 0.03) |
| WSH | 580 | -0.88 | 0.09 (-0.03, 0.21) | 0.08 (-0.03, 0.19) |
| **Head circumference-for-age Z-score** | | | | |
| Nutrition + WSH | 590 | -1.51 | | |
| Nutrition | 570 | -1.45 | -0.05 (-0.17, 0.07) | -0.04 (-0.15, 0.08) |
| WSH | 584 | -1.59 | 0.08 (-0.04, 0.19) | 0.09 (-0.01, 0.20) |

*Adjusted for pre-specified covariates using targeted maximum likelihood estimation with data-adaptive model selection [Arnold 2013, Balzer 2016]: Field staff who collected data, month of measurement, household food insecurity, child age, child sex, mother's age, mothers height, mothers education level, number of children < 18 years in the household, number of individuals living in the compound, distance in minutes to the primary water source, household roof, floor, wall materials, household assets.

## Table S5: Proportion of children stunted, severely stunted, wasted and underweight after 2 years of intervention – single and combined intervention arms minus control arm unadjusted and adjusted analyses

| | | | Difference from control (95% CI) | |
|---|---|---|---|---|
| **Outcome, Arm** | **N** | **Prevalence** | **Unadjusted*** | **Adjusted†** |
| **Stunting** | | | | |
| Control | 1103 | 40.9% | | |
| Water | 595 | 42.9% | 2.4 (-2.6, 7.3) | 1.0 (-4.1, 6.1) |
| Sanitation | 579 | 40.1% | -0.4 (-5.3, 4.6) | -2.4 (-7.3, 2.5) |
| Handwashing | 570 | 46.1% | 5.3 (0.2, 10.3) | 4.1 (-0.9, 9.1) |
| Water + Sanitation + Handwashing (WSH) | 579 | 40.1% | -0.5 (-5.5, 4.4) | -1.7 (-6.4, 3.0) |
| Nutrition | 567 | 32.8% | -7.7 (-12.4, -2.9) | -8.6 (-13.1, -4.2) |
| Nutrition + WSH | 591 | 37.4% | -3.8 (-8.6, 1.1) | -6.0 (-10.5, -1.5) |
| **Severe stunting** | | | | |
| Control | 1103 | 11.2% | | |
| Water | 595 | 14.5% | 3.3 (-0.1, 6.7) | 2.4 (-0.8, 5.6) |
| Sanitation | 579 | 11.2% | 0.1 (-3.0, 3.3) | -1.1 (-4.5, 2.3) |
| Handwashing | 570 | 11.4% | 0.2 (-3.0, 3.4) | -0.4 (-3.3, 2.6) |
| Water + Sanitation + Handwashing (WSH) | 579 | 10.2% | -1.0 (-4.1, 2.1) | -1.5 (-4.6, 1.5) |
| Nutrition | 567 | 8.3% | -2.8 (-5.7, 0.2) | -3.3 (-6.1, -0.6) |
| Nutrition + WSH | 591 | 8.5% | -3.0 (-5.9, -0.0) | -3.4 (-6.2, -0.5) |
| **Wasting** | | | | |
| Control | 1104 | 10.7% | | |
| Water | 596 | 12.2% | 1.8 (-1.4, 5.0) | 1.6 (-2.1, 5.2) |
| Sanitation | 580 | 11.2% | 0.9 (-2.3, 4.0) | 0.2 (-2.9, 3.4) |
| Handwashing | 570 | 10.5% | 0.1 (-3.1, 3.2) | -0.3 (-3.3, 2.7) |
| Water + Sanitation + Handwashing (WSH) | 580 | 11.9% | 1.4 (-1.8, 4.6) | 1.4 (-2.1, 4.8) |
| Nutrition | 567 | 8.8% | -1.6 (-4.5, 1.3) | -2.1 (-4.8, 0.7) |
| Nutrition + WSH | 591 | 8.8% | -1.7 (-4.7, 1.2) | -1.9 (-4.4, 0.7) |
| **Underweight** | | | | |
| Control | 1121 | 30.7% | | |
| Water | 599 | 35.6% | 5.3 (0.7, 10.0) | 4.1 (-0.5, 8.7) |
| Sanitation | 588 | 30.4% | 0.3 (-4.3, 4.9) | -1.7 (-6.1, 2.6) |
| Handwashing | 573 | 34.4% | 3.9 (-0.9, 8.7) | 2.7 (-2.5, 7.9) |
| Water + Sanitation + Handwashing (WSH) | 586 | 32.8% | 2.2 (-2.4, 6.8) | 1.6 (-2.7, 5.8) |
| Nutrition | 573 | 26.0% | -4.2 (-8.6, 0.3) | -5.1 (-9.1, -1.1) |
| Nutrition + WSH | 592 | 25.0% | -5.8 (-10.2, -1.4) | -6.5 (-10.9, -2.2) |

*Unadjusted estimates were estimated using a pair-matched Mantel-Haenszel analysis.

† Adjusted for pre-specified covariates using targeted maximum likelihood estimation with data-adaptive model selection [Arnold 2013, Balzer 2016]: Field staff who collected data, month of measurement, household food insecurity, child age, child sex, mother's age, mothers height, mothers education level, number of children < 18 years in the household, number of individuals living in the compound, distance in minutes to the primary water source, household roof, floor, wall materials, household assets.

## Table S6: Proportion of children stunted, severely stunted, wasted and underweight after 2 years of intervention – Nutrition + WSH arm minus Nutrition and WSH arms unadjusted and adjusted analyses

| | | | Difference from control (95% CI) | |
|---|---|---|---|---|
| | **N** | **Prevalence** | **Unadjusted*** | **Adjusted†** |
| **Stunting** | | | | |
| Nutrition + WSH | 591 | 37.4% | | |
| Nutrition | 567 | 32.8% | 4.0 (-1.6, 9.6) | 3.3 (-2.2, 8.9) |
| WSH | 579 | 40.1% | -2.8 (-8.4, 2.8) | -4.4 (-9.4, 0.7) |
| **Severe stunting** | | | | |
| Nutrition + WSH | 591 | 8.5% | | |
| Nutrition | 567 | 8.3% | -0.3 (-3.5, 3.0) | 0.3 (-2.8, 3.4) |
| WSH | 579 | 10.2% | -1.9 (-5.2, 1.4) | -2.0 (-5.2, 1.2) |
| **Wasting** | | | | |
| Nutrition + WSH | 591 | 8.8% | | |
| Nutrition | 567 | 8.8% | 0.2 (-3.0, 3.5) | 0.5 (-2.3, 3.2) |
| WSH | 580 | 11.9% | -2.8 (-6.3, 0.7) | -2.8 (-6.3, 0.6) |
| **Underweight** | | | | |
| Nutrition + WSH | 592 | 25.0% | | |
| Nutrition | 573 | 26.0% | -1.7 (-6.6, 3.3) | -1.1 (-5.5, 3.2) |
| WSH | 586 | 32.8% | -7.8 (-12.9, -2.6) | -8.4 (-13.4, -3.4) |

*Unadjusted estimates were estimated using a pair-matched Mantel-Haenszel analysis.

† Adjusted for pre-specified covariates using targeted maximum likelihood estimation with data-adaptive model selection [Arnold 2013, Balzer 2016]: Field staff who collected data, month of measurement, house- hold food insecurity, child age, child sex, mother's age, mothers height, mothers education level, number of children < 18 years in the household, number of individuals living in the compound, distance in minutes to the primary water source, household roof, floor, wall materials, household assets.

## Table S7: All-cause mortality among index children 2 years after intervention

| Arm | N at risk* | n deaths | Cumulative incidence | Risk difference† (95% CI) |
|---|---|---|---|---|
| Control | 1306 | 62 | 0.047 | |
| Water | 656 | 27 | 0.041 | -0.006 (-0.025, 0.013) |
| Sanitation | 652 | 27 | 0.041 | -0.005 (-0.024, 0.014) |
| Handwashing | 648 | 29 | 0.045 | -0.003 (-0.023, 0.017) |
| WSH | 662 | 31 | 0.047 | -0.000 (-0.020, 0.019) |
| Nutrition | 651 | 25 | 0.038 | -0.009 (-0.027, 0.010) |
| Nutrition + WSH | 646 | 19 | 0.029 | -0.018 (-0.036, -0.001) |

* Number of live born infants

† Unadjusted estimates were estimated using a pair-matched Mantel-Haenszel analysis.

## Table S8: Between-cluster spillovers conditional on distance to the nearest treated compound - Unadjusted permutation test p-values

| Arm | $N_{20}$[a] | $N_{80}$[b] | $Mean_{20}$[c] | $Mean_{80}$[d] | Difference[e] | P-value[f] |
|---|---|---|---|---|---|---|
| **Length-for-age z-score** | | | | | | |
| Water | 221 | 221 | -1.743 | -1.799 | -0.056 | 0.565 |
| Sanitation | 221 | 221 | -1.785 | -1.753 | 0.032 | 0.733 |
| Handwashing | 221 | 221 | -1.735 | -1.796 | -0.061 | 0.520 |
| WSH | 221 | 221 | -1.843 | -1.711 | 0.132 | 0.171 |
| Nutrition | 221 | 221 | -1.765 | -1.748 | 0.017 | 0.862 |
| Nutrition+WSH | 221 | 221 | -1.738 | -1.796 | -0.057 | 0.556 |
| **Diarrhea** | | | | | | |
| Water | 704 | 707 | 0.070 | 0.054 | -0.016 | 0.195 |
| Sanitation | 705 | 704 | 0.058 | 0.055 | -0.003 | 0.836 |
| Handwashing | 705 | 707 | 0.061 | 0.057 | -0.004 | 0.698 |
| WSH | 703 | 704 | 0.055 | 0.084 | 0.028 | 0.024 |
| Nutrition | 705 | 704 | 0.060 | 0.057 | -0.003 | 0.849 |
| Nutrition+WSH | 707 | 703 | 0.072 | 0.055 | -0.017 | 0.184 |

[a] $N_{20}$ = the number of control compounds with a distance to the nearest treated compound less than or equal to the 20th percentile of the observed distribution of distance
[b] $N_{80}$ = the number of control compounds with a distance to the nearest treated compound greater than or equal to the 80th percentile of the observed distribution of distance
[c] $Mean_{20}$ = the mean outcome in control compounds with a distance to the nearest treated compound less than or equal to the 20th percentile of the observed distribution of distance
[d] $Mean_{80}$ = the mean outcome in control compounds with a distance to the nearest treated compound greater than or equal to the 80th percentile of the observed distribution of distance
[e] Difference = $Mean_{80}$ - $Mean_{20}$
[f] Permutation test p-value

## Table S9: Between-cluster spillovers conditional on distance to the nearest treated compound - Adjusted permutation test p-values

| Arm | $N_{20}$[a] | $N_{80}$[b] | $Mean_{20}$[c] | $Mean_{80}$[d] | Difference[e] | P-value[f] |
|---|---|---|---|---|---|---|
| **Length-for-age z-score** | | | | | | |
| Water | 220 | 220 | -1.743 | -1.799 | -0.045 | 0.600 |
| Sanitation | 220 | 220 | -1.785 | -1.753 | 0.018 | 0.831 |
| Handwashing | 220 | 220 | -1.735 | -1.796 | -0.051 | 0.548 |
| WSH | 220 | 220 | -1.843 | -1.711 | 0.108 | 0.204 |
| Nutrition | 220 | 220 | -1.765 | -1.748 | -0.037 | 0.665 |
| Nutrition+WSH | 220 | 220 | -1.738 | -1.796 | -0.052 | 0.538 |
| **Diarrhea** | | | | | | |
| Water | 612 | 612 | 0.070 | 0.054 | -0.023 | 0.090 |
| Sanitation | 615 | 611 | 0.058 | 0.055 | -0.008 | 0.564 |
| Handwashing | 612 | 613 | 0.061 | 0.057 | -0.002 | 0.898 |
| WSH | 612 | 615 | 0.055 | 0.084 | 0.029 | 0.032 |
| Nutrition | 611 | 611 | 0.060 | 0.057 | -0.004 | 0.763 |
| Nutrition+WSH | 612 | 613 | 0.072 | 0.055 | -0.025 | 0.062 |

[a] $N_{20}$ = the number of control compounds with a distance to the nearest treated compound less than or equal to the 20th percentile of the observed distribution of distance

[b] $N_{80}$ = the number of control compounds with a distance to the nearest treated compound greater than or equal to the 80th percentile of the observed distribution of distance

[c] $Mean_{20}$ = the mean outcome in control compounds with a distance to the nearest treated compound less than or equal to the 20th percentile of the observed distribution of distance

[d] $Mean_{80}$ = the mean outcome in control compounds with a distance to the nearest treated compound greater than or equal to the 80th percentile of the observed distribution of distance

[e] Adjusted difference = $Mean_{80}$ - $Mean_{20}$. Models adjusted for pre-specified covariates using with data-adaptive model selection [Arnold 2013]: Field staff who collected data, month of measurement, household food insecurity, child age, child sex, mother’s age, mother’s height, mother’s education level, number of children < 18 years in the household, number of individuals living in the compound, distance in minutes to the primary water source, household roof, floor, wall materials, household assets.

[f] Permutation test p-value

## Table S10: Between-cluster spillovers on length-for-age z-score through shared institutions - Unadjusted permutation test p-values

| Arm | Shared market | | Shared health clinic | | Shared mosque/church | |
|---|---|---|---|---|---|---|
| | Difference[a] | p-value[b] | Difference[a] | p-value[b] | Difference[a] | p-value[b] |
| **Length-for-age z-score** | | | | | | |
| Water | 0.046 | 0.467 | 0.107 | 0.259 | 0.070 | 0.605 |
| Sanitation | 0.056 | 0.388 | 0.07 | 0.438 | -0.045 | 0.713 |
| Handwashing | -0.022 | 0.739 | -0.003 | 0.975 | -0.165 | 0.215 |
| WSH | -0.062 | 0.36 | -0.184 | 0.064 | -0.232 | 0.135 |
| Nutrition | -0.084 | 0.217 | -0.099 | 0.296 | -0.006 | 0.959 |
| Nutrition+WSH | 0.03 | 0.643 | -0.08 | 0.379 | -0.054 | 0.703 |
| **Diarrhea** | | | | | | |
| Water | -0.008 | 0.374 | -0.015 | 0.222 | -0.005 | 0.657 |
| Sanitation | 0.003 | 0.687 | -0.016 | 0.171 | -0.015 | 0.274 |
| Handwashing | 0.014 | 0.128 | -0.006 | 0.564 | -0.026 | 0.112 |
| WSH | -0.016 | 0.073 | -0.005 | 0.713 | 0.002 | 0.869 |
| Nutrition | -0.005 | 0.565 | -0.023 | 0.071 | -0.014 | 0.389 |
| Nutrition+WSH | -0.007 | 0.427 | 0.007 | 0.570 | -0.011 | 0.494 |

[a] Difference in the mean outcome among control compounds that shared an institution with a treated com- pound and those that did not
[b] Permutation test p-value

## Table S11: Between-cluster spillovers on length-for-age z-score through shared institutions - Adjusted permutation test p-values

| Arm | Shared market | | Shared health clinic | | Shared mosque/church | |
|---|---|---|---|---|---|---|
| | Difference[a] | p-value[b] | Difference[a] | p-value[b] | Difference[a] | p-value[b] |
| **Length-for-age z-score** | | | | | | |
| Water | 0.028 | 0.626 | 0.09 | 0.288 | 0.086 | 0.463 |
| Sanitation | 0.017 | 0.763 | 0.037 | 0.642 | -0.036 | 0.728 |
| Handwashing | -0.006 | 0.914 | 0.015 | 0.854 | -0.105 | 0.364 |
| WSH | -0.025 | 0.67 | -0.138 | 0.113 | -0.204 | 0.142 |
| Nutrition | -0.077 | 0.201 | -0.04 | 0.631 | -0.008 | 0.945 |
| Nutrition+WSH | 0.022 | 0.697 | -0.072 | 0.374 | 0.011 | 0.934 |
| **Diarrhea** | | | | | | |
| Water | -0.007 | 0.41 | -0.011 | 0.441 | 0.002 | 0.888 |
| Sanitation | 0.007 | 0.433 | -0.01 | 0.43 | -0.012 | 0.436 |
| Handwashing | 0.012 | 0.175 | -0.006 | 0.649 | -0.027 | 0.118 |
| WSH | -0.013 | 0.151 | -0.001 | 0.959 | 0.001 | 0.973 |
| Nutrition | -0.006 | 0.508 | -0.021 | 0.122 | -0.01 | 0.549 |
| Nutrition+WSH | -0.005 | 0.612 | 0.008 | 0.543 | -0.012 | 0.523 |

[a] Adjusted difference in mean outcome among control compounds that shared an institution with a treated compound and those that did not. Models adjusted for pre-specified covariates using with data-adaptive model selection [Arnold 2013]: Field staff who collected data, month of measurement, household food insecurity, child age, child sex, mother's age, mother's height, mother's education level, number of children < 18 years in the household, number of individuals living in the compound, distance in minutes to the primary water source, household roof, floor, wall materials, household assets.
[b] Permutation test p-value

## Inclusion / Exclusion criteria—WASH Benefits Bangladesh protocol excerpt

Compounds (within eligible communities) will be eligible to participate if they include at least one pregnant women in the first two trimesters of her pregnancy who is currently living in the compound. Within each enrolled compound, we will collect information from three types of children:

(1) Infants (target child) will be eligible to participate in the study if they are:

1. They were in utero at the baseline survey
2. Their parents/guardians are planning to stay in the study village for the next 24months (if a mother is planning to give birth at her natal home and then return, she will still be a candidate for enrollment)

(2) Children < 36 months at baseline that are living in the compound of a target child will be eligible to participate in diarrhea measurement if:

1. They are 3 – 36 months old at the baseline survey
2. Their parents/guardians are planning to stay in the study village for the next 12 months

(3) In addition to the target child, up to two older siblings or older children from each enrolled compound that includes a target child will be eligible to participate in the intestinal parasite specimen measurement if they are 3.5 to 12 years old. Specifically, these children will include the following:

1. Children between the ages of 3.5 and 5: These children were enrolled for specimen at baseline and at that time they were are between the ages of 18-27 months. Parasite assessment in these children will be done at both baseline and endline.
2. Children between the ages of 5-12 years: Parasite assessment in these children will only be done at endline.
3. Their parents/guardians are planning to stay in the study village for the next 12 months after baseline enrollment.

There will be no exclusion criteria based on gender, race, or ethnicity.

Compounds will be eligible to participate in the spillover substudy if they are located in close proximity (within 2 minutes walking distance) to enrolled compounds in the combined intervention and control arms and if a child 0-59 months resides there at endline.

## Outcome definitions – WASH Benefits Bangladesh excerpt

Primary Outcomes

- Length-for-age Z-scores (HAZ)after 2 years of intervention
- Caregiver-reported diarrhea (3 or more loose stools in a 24 hour period during the last 7 days)

Secondary Outcomes

- HAZ measured 1 year after intervention
- Ages and Stages Questionnaire (ASQ) child development scores (motor, communication, personal/social) after 2 years of intervention
- Verbal Communicative Development Inventory (CDI) scores at 2 year follow-up
- Executive function scores at 2 year follow-up (A-NOT-B task and Tower Test)
- Proportion of children stunted (HAZ < – 2) 2 years after intervention
- Enteropathy biomarker measurements at 1 and 2 years after intervention
    - Lactulose / mannitol sugar permeability test
    - Antibody titers (Total IgG)
- Mean hemoglobin measurements at 2 years after intervention

Tertiary Outcomes

- Weight-for-age Z-scores at 1 and 2 years after intervention
- Weight-for-height Z-scores at 1 and 2 years after intervention
- Proportion of children underweight (WAZ < – 2) after 2 years of intervention
- Proportion of children wasted (WHZ < – 2) after 2 years of intervention
- Proportion of children severely stunted (HAZ < – 3) after 2 years of intervention
- Head circumference Z-scores after 2 years of intervention
- Parasitic infections at 2 year follow-up
    - Soil transmitted helminths (Ascaris, Trichuris, Hookworm)
    - Protozoans (Giardia, Cryptosporidium, E. histolytica)
- Verbal Communicative Development Inventory (CDI)scores at 1 year follow-up
- WHO Motor milestones at 1 year follow-up
- Caregiver reported respiratory illness in the preceding 7 days
- Degree of exposure to interventions
- Behavioral response to interventions
- Mortality
- Proportion of children with anemia (Hb<110 g/L)
- Micronutrient status at 2 years after intervention (iron, vitamin A, folate, B12)

After a mother has been enrolled in the study, trained icddr,b staff will conduct a baseline assessment. Mothers will be asked standard questions about their (and spousal) education, activities, occupation, household assets, and current sanitation & hygiene practices. The baseline assessment in all participating households will include bar soap and detergent powder consumption measurements, latrine use (visual inspection), and numerous spot-check hygiene indicators (e.g., presence of animal or human feces in the household environment). The field team will collect these measurements at the three measurement rounds of the study (baseline, 1-year follow-up, 2-year follow-up).

Monitoring Of Uptake:
At each quarterly visit, we will monitor the presence of functional latrine and faeces disposal hardware, latrine use and faeces disposal practices and presence and quantity of feces in the living environment using spot check observations. We propose collecting repeated measures because spot check observations are a noisy indicator of household behaviors because of temporal variation, and a longitudinal index based on repeated measurements can more finely distinguish meaningful behavioral patterns [Gorter 1998, Ruel 2002]. We also expect time trends in the uptake of the latrine intervention; uptake might initially be low due to unfamiliarity, increase with behavior change promotion and taper off as the novelty dissipates. Quarterly data will allow us to monitor these trends.

Monitoring of Child Health Outcomes:

At each visit, we will also administer a brief interview to the primary caregiver of the index child to assess caregiver reported diarrhea for the index child and other children < 60 months living in the compound.

Diarrhoea, other symptoms of illness and negative control outcomes were collected using the following questionnaire (translated to Bengali) (The full illness assessment module is available at osf.io/gejux):

# WASH Benefits Bangladesh Common Module 2
# Diarrhea and symptoms of illness

Respondent: Child's primary caregiver.
(The primary caregiver is the person that spends the most time with the child. This is often the mother.)

| **IDENTIFICATION** | |
|---|---|
| **0.1. CLUSTER ID:** | ☐☐☐☐ |
| **0.2. HOUSEHOLD ID:** | ☐☐ |
| **0.3. CHILD ID** | ☐☐☐☐☐☐ |

| | **A** | **B** | **C** | **D** |
|---|---|---|---|---|
| **Did [NAME] have [SYMPTOM] :** | **Today** | **Yesterday** | **Day before Yesterday** | **In the last 7 days** (since this day last week) |
| **C.201** Fever | 1=Yes | 1=Yes | 1=Yes | 1=Yes |
| **C.202** Diarrhea | 1=Yes | 1=Yes | 1=Yes | 1=Yes |
| **C.203** 3 or more bowel movements in 24 hours | 1=Yes | 1=Yes | 1=Yes | 1=Yes |
| **C.204** Number of bowl movements each day | | | | |
| **C.205** Watery or soft stool (unformed) | 1=Yes | 1=Yes | 1=Yes | 1=Yes |
| **C.206** Blood in the stool | 1=Yes | 1=Yes | 1=Yes | 1=Yes |
| **C.207** Skin rash (anywhere on the body) | 1=Yes | 1=Yes | 1=Yes | 1=Yes |
| | **Today** | **Yesterday** | **Day before Yesterday** | **In the last 7 days** (since this day last week) |
| **C.208** Constant cough | 1=Yes | 1=Yes | 1=Yes | 1=Yes |
| **C.209** Congestion / runny nose | 1=Yes | 1=Yes | 1=Yes | 1=Yes |
| **C.210** Panting / wheezing / difficulty breathing | 1=Yes | 1=Yes | 1=Yes | 1=Yes |
| **C.211** Bruising, scrapes or cuts | 1=Yes | 1=Yes | 1=Yes | 1=Yes |
| **C.212** Toothache / teething | 1=Yes | 1=Yes | 1=Yes | 1=Yes |

**C.213**
*If answered Yes to C.202 (Diarrhea):* When did the diarrhea start?
Record length of time in days or weeks. If< 14 days, record the response in days.

A ☐☐ B ☐

1 Days ago
2 Weeks ago

Anthropometry Assessment

Field teams will measure outcomes at 1 year following the initiation of intervention. Children will be between 8and 15months at the 1-year survey. This will be the first round in which the field team measures anthropometry. The team will collect information about how many weeks the children and their mothers stayed in another village to understand how many weeks they were out of interventions. The field teams will measure length, weight, and head circumference using standardized measurement techniques. Our anthropometric teams will have been trained and standardized in measurement techniques according to the FANTA and WHO guidelines (Cogill 2003, deOnis 2004). The child will be weighed using a calibrated scale and measure his/her length or height using a height board and head circumference using a tape measure. We will also measure maternal height and weight.

(The full anthropometry assessment module is available at osf.io/gejux)

## WASH Benefits Bangladesh Common Module 4 Anthropometry

Administer to: Children at midline and endline

| **IDENTIFICATION** | |
|---|---|
| **0.1. CLUSTER ID:** | ☐☐☐☐ |
| **0.2. HOUSEHOLD ID:** | ☐☐☐ |
| **0.3. CHILD ID** | ☐☐☐☐☐☐ |

| | | |
|---|---|---|
| **C.401**<br>**FRA ID** | ## | ☐☐ |
| **C.402**<br>**Name of FRA** | Full Name | |
| **C.403**<br>**Is mother wearing heavy clothing during weight measurement?** | 1 = Light clothing<br>2 = Light clothing plus sweater<br>3 = Heavy clothing | ☐ |
| **C.404**<br>**Weight of Mother Measurement #1** | Weight (kg) | ☐☐.☐ |

| | | |
|---|---|---|
| **C.405**<br>**Weight of Mother**<br>**Measurement #2** | Weight<br>(kg) | □□.□ |
| **C.406**<br>**Weight of Mother**<br>**Measurement #3**<br>(If difference between measures 1 & 2 is ≥ 0.1 kg) | Weight<br>(kg) | □□.□ |
| **C.407**<br>**Is child wearing clothing during weight measurement?** | 0 = No Clothes<br>1 = Only Shirt<br>2 = Only Pants<br>3 = Both Shirt & Pants | □ |
| **C.408**<br>**Weight of Mother + Child**<br>**Measurement #1** | Weight<br>(kg) | □□.□ |
| **C.409**<br>**Weight of Mother + Child**<br>**Measurement #2** | Weight<br>(kg) | □□.□ |
| **C.410**<br>**Weight of Mother + Child**<br>**Measurement #3**<br>(If difference between measures 1 & 2 is ≥ 0.1 kg) | Weight<br>(kg) | □□.□ |
| **C.411 – C.413**<br>reserved for child weight measurement without mother (follow-up visits) | | |
| **C.414**<br>**Length of Child**<br>**Measurement #1** | Length<br>(cm) | □□□.□ |
| **C.415**<br>**Length of Child**<br>**Measurement #2** | Length<br>(cm) | □□□.□ |

| | | |
|---|---|---|
| **C.416**<br>**Length of Child**<br>**Measurement #3**<br>(If difference between measures 1 & 2 is ≥ 0.5 cm) | Length<br>(cm) | □□□.□ |
| **C.417**<br>**Length Measurement Method** | Child was:<br><br>1 = lying (recumbent)<br>2 = standing | □ |
| **C.418**<br>**Head Circumference**<br>**Measurement #1** | Circumference<br>(cm) | □□.□ |
| **C.419**<br>**Head Circumference**<br>**Measurement #2** | Circumference<br>(cm) | □□.□ |
| **C.420**<br>**Head Circumference**<br>**Measurement #3**<br>(If difference between measures 1 & 2 is ≥ 0.5 cm) | Circumference<br>(cm) | □□.□ |
| **C.421**<br>**Does the child have swollen feet (bi-pedal edema)?** | 1 = Yes (**>> Referral**)<br>2 = No | □ |
